# Iron Oxide Nanoparticles Engineered Macrophage-Derived Exosomes for Targeted Pathological Angiogenesis Therapy

**DOI:** 10.1021/acsnano.4c00699

**Published:** 2024-02-27

**Authors:** Haorui Zhang, Yu Mao, Zheng Nie, Qing Li, Mengzhu Wang, Chang Cai, Weiju Hao, Xi Shen, Ning Gu, Wei Shen, Hongyuan Song

**Affiliations:** # Department of Ophthalmology, Shanghai Changhai Hospital, Shanghai 200433, P.R. China; ‡ Nanjing Key Laboratory for Cardiovascular Information and Health Engineering Medicine, Institute of Clinical Medicine, Nanjing Drum Tower Hospital, Medical School, Nanjing University, Nanjing 210093, P.R. China; § University of Shanghai for Science and Technology, Shanghai 200093, P.R. China; ∥ Department of Ophthalmology, Ruijin Hospital, Shanghai Jiao Tong University School of Medicine, Shanghai 200020, P.R. China

**Keywords:** engineered exosomes, ferroptosis, immunotherapy, macrophage, pathological angiogenesis, retinopathy

## Abstract

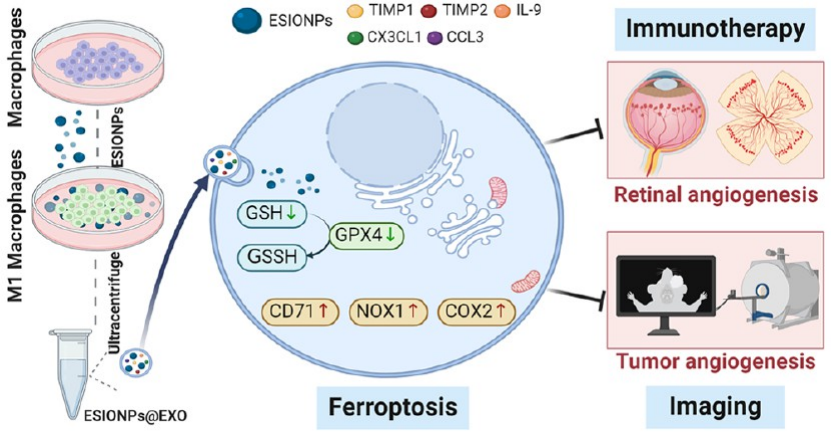

Engineering exosomes with nanomaterials usually leads to the damage of exosomal membrane and bioactive molecules. Here, pathological angiogenesis targeting exosomes with magnetic imaging, ferroptosis inducing, and immunotherapeutic properties is fabricated using a simple coincubation method with macrophages being the bioreactor. Extremely small iron oxide nanoparticle (ESIONPs) incorporated exosomes (ESIONPs@EXO) are acquired by sorting the secreted exosomes from M1-polarized macrophages induced by ESIONPs. ESIONPs@EXO suppress pathological angiogenesis *in vitro* and *in vivo* without toxicity. Furthermore, ESIONPs@EXO target pathological angiogenesis and exhibit an excellent T1-weighted contrast property for magnetic resonance imaging. Mechanistically, ESIONPs@EXO induce ferroptosis and exhibit immunotherapeutic ability toward pathological angiogenesis. These findings demonstrate that a pure biological method engineered ESIONPs@EXO using macrophages shows potential for targeted pathological angiogenesis therapy.

Exosomes, with a size range of 50–150 nm, mediate the cross-talk between different cells.^1^ They are emerging as ideal vesicles for drug delivery, diagnosis, immunotherapy, and precision medicine.^2^ Engineering exosomes with nanomaterials is extensively studied to increase the therapeutic efficiency and decrease the toxicity of nanomaterials.^3−6^ However, the current engineering methods (e.g., sonication or electroporation) cannot avoid damaging exosomal membrane and bioactive molecule inside.^7−9^ In the present work, the concept of cell bioreactor assisted exosome modification with nanomaterials is proposed. Exosomes derived from different cells usually exhibit different therapeutic functions. This engineering method utilizes the bioactive properties of exosomes and specific characteristics of nanomaterials, which will exhibit great potential in therapeutics.

Iron oxide nanoparticles (IONPs) are usually used as imaging reagents and nanocarriers for target therapy because of magnetic properties.^10^ It is reported that IONPs can stimulate mesenchymal stem cells (MSCs) to express therapeutic growth factor to attenuate ischemic stroke and enhance cardiac repair.^11,12^ Extracellular nanovesicles derived from IONP-incorporated MSCs are developed via extrusion to strengthen their efficiency by magnetic guidance.^11^ However, the intrinsic therapeutic effect of engineered exosomes secreted from IONP-treated cells is still elusive. Macrophage-derived exosomes are reported to participate in multiple biological processes.^13^ The exosomes derived from various types of macrophages show completely different activity.^13^ M2 macrophage-derived exosomes can facilitate angiogenesis and tumor growth,^14^ whereas M1 macrophage-derived exosomes exhibit antiangiogenic and antitumor activity.^15,16^ Importantly, M1 macrophage-derived exosomes are able to repolarize M2 macrophages to M1 macrophages.^17^ These studies indicate a vital role of macrophage-derived exosomes in immunotherapy. IONPs have been reported to induce M1 macrophage polarization to potentiate macrophage-modulating cancer immunotherapies.^18^ M1 macrophage-derived nanovesicles are reported to suppress angiogenesis.^13^ However, the intrinsic therapeutic effect of exosomes derived from IONP-treated macrophages is largely unknown. It is speculated that IONP-induced M1 macrophage-derived exosomes exhibit immunotherapeutic function for pathological angiogenesis.

Blood vessels connect to all tissues to sustain vital movement. Different from physiological angiogenesis, pathological angiogenesis is usually highly permeable, with abnormal shape and dysfunctionality.^19^ The occurrence of pathological angiogenesis contributes to tumor, retinopathies, rheumatoid arthritis, cardiovascular diseases, etc.^19^ Antivascular endothelial growth factor (anti-VEGF) reagents have shown potential in pathological angiogenesis therapy.^20^ However, drug resistance, repeated treatment, and systematic adverse effects still need to be addressed in many patients. Due to their specific physiochemical properties, nanosized drugs have attracted extensive attention.^21^ Among them, nanomaterial-engineered exosomes exhibit excellent biocompatibility and therapeutic efficiency.

Iron-based nanoparticles have been reported to induce ferroptosis in multiple tissues.^22−24^ Of them, IONPs are extensively studied as they have been approved for clinical application and are biocompatible material.^18^ Recently, IONPs are used to engineer extracellular vesicles for combined therapy with specific functions such as magnetic guidance, imaging, and ferroptosis induction.^5^ The relatively large size of IONPs affects the intrinsic bioactive molecules of exosomes, which restricts their further application.^25^ Extremely small sized iron oxide nanoparticles (ESIONPs) with sizes less than 5 nm function as T1 contrast agents for magnetic resonance imaging (MRI).^26^ They exhibit good efficiency for ferroptosis induction;^27^ however, the response of the immune system cannot be ignored.^28^ Exosomes are well studied carriers to improve tissue targeting ability and reduce the toxicity of nanomaterials,^2^ and delivering ESIONPs via exosomes may be a good option. Combining the immunotherapeutic properties of M1 macrophage-derived exosomes with ferroptosis-inducing roles of ESIONPs could be promising for the suppression of aberrant angiogenesis.

In this study, the concept of cell bioreactor assisted exosome modification is proposed to construct exosome-incorporated ESIONPs (ESIONPs@EXO). This is a pure natural biological process without any damage to the engineered exosomes. ESIONPs@EXO exhibit pathological angiogenesis targeting, magnetic imaging, ferroptosis inducing, and immunotherapeutic properties (Figure 1). ESIONPs@EXO could suppress vascular endothelial cells (ECs) angiogenic roles *in vitro*. Following the injection of ESIONPs@EXO, the retention of ESIONPs@EXO was found to be the result of pathological angiogenesis. As a result, ESIONPs@EXO attenuated pathological retinal angiogenesis and suppressed tumor angiogenesis and tumor growth. Mechanistically, ESIONPs@EXO induced ferroptosis and exhibited an immunotherapeutic ability. Overall, ESIONPs@EXO could be a biocompatible nanoplatform for pathological angiogenesis imaging and therapy.

**Figure 1:**
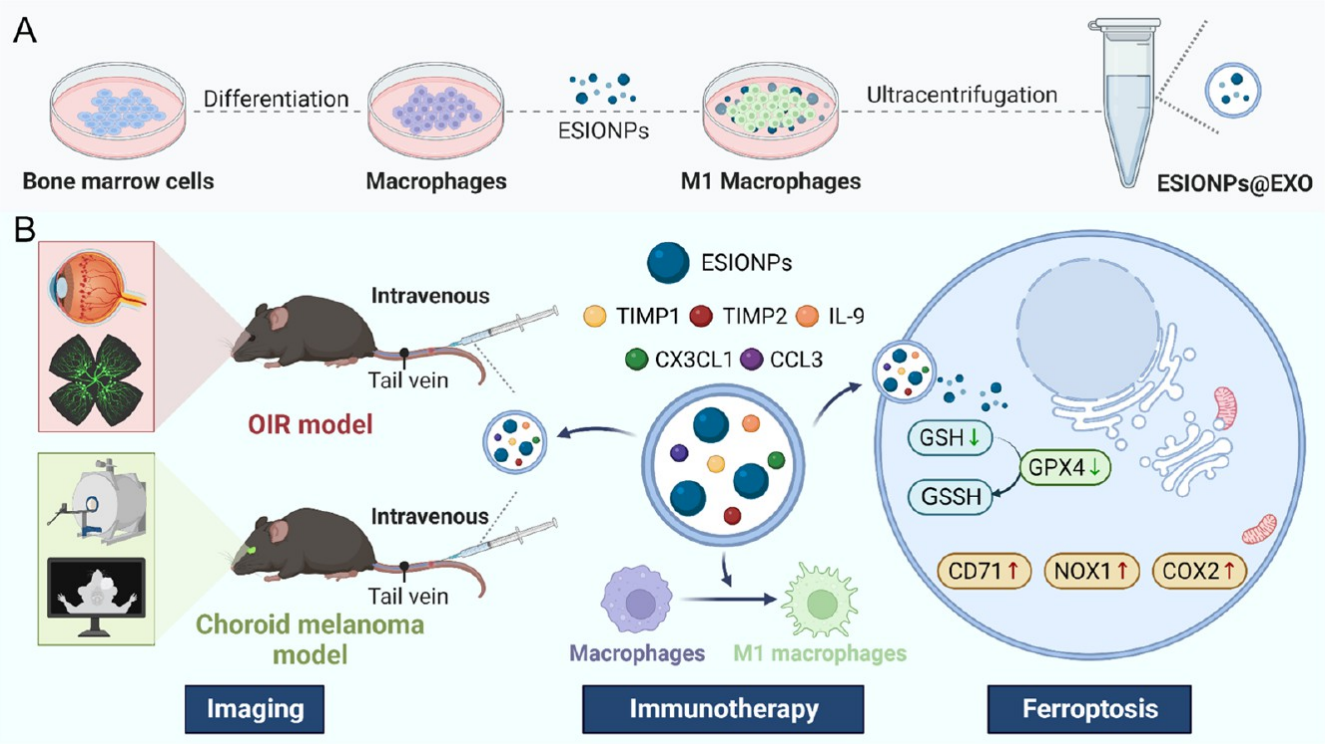
Illustration of ESIONPs@EXO preparation (A) and targeted pathological angiogenesis therapy (B).

## Results and Discussion

### Characterization of ESIONPs@EXO

Engineering exosomes with nanoparticles has shown therapeutic potential in tumor treatment. However, damage to the membrane and bioactive molecules of exosomes is hardly avoided.^7−9^ Recent studies indicate that nanomaterials affect the status of the cell and alter the bioactivities of cell-secreted exosomes apparently.^11,12,29^ We synthesized ESIONPs via a fluidic reactor as previous reported^26^ and assessed the exosomes derived from macrophages treated with ESIONPs. ESIONPs with a size of 3.7 nm were used in the present study (Figure S1). Different from other cells, macrophages are characterized by high activity in phagocytosis and exocytosis.^30^ Several reports have shown that nanomaterials are easily internalized and secreted by macrophages, which make them ideal bioreactors for engineering.^31−33^ Then, we used two types of macrophages (bone marrow derived macrophages, BMMs, and RAW 264.7) to study the effect of ESIONPs on macrophages. These ESIONPs could induce M1 macrophage polarization in both macrophages without affecting their viability (Figures S2–S4). Tumor necrosis factor alpha (TNF-α) is the key marker for M1 macrophages.^18^ The results showed that ESIONPs treatment increased TNF-α levels dose dependently (Figure S3), whereas ESIONPs did not further increase TNF-α levels with a concentration higher than 250 μg/mL. Thus, 250 μg/mL of ESIONPs was used to coincubate with cells in the study. As the murine-leukemic monocyte-macrophage cell line, the phenotype of RAW 264.7 could change with continuous culture.^34^ Therefore, primary BMMs were used for engineering ESIONPs@EXO in subsequent experiments.

**Figure 2:**
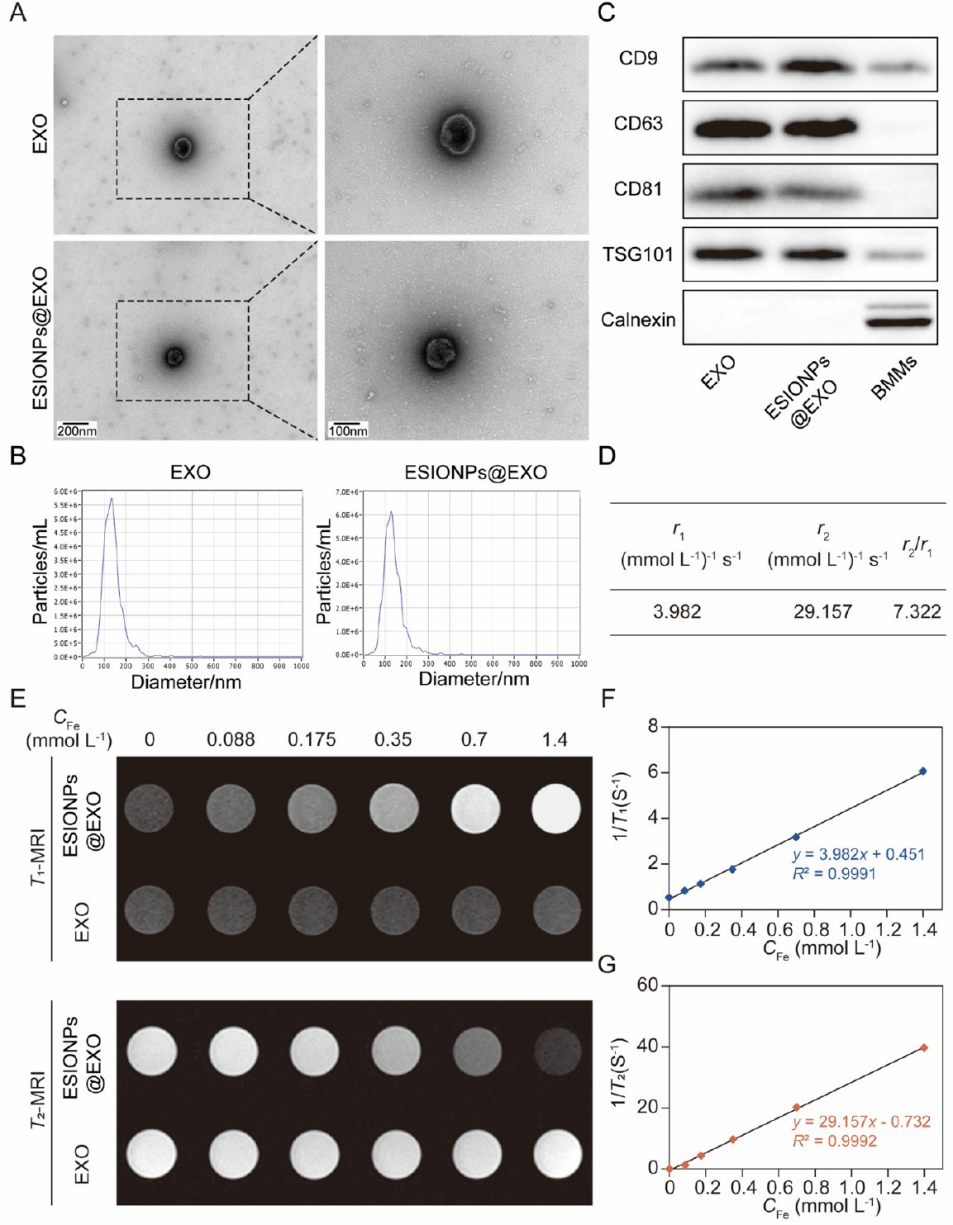
Characterization of ESIONPs@EXO derived from ESIONPs engineered macrophages. (A) The morphology of EXO and ESIONPs@EXO determined by TEM. Scale bar: 200 nm (left) and 100 nm (right). (B) The size distribution of EXO and ESIONPs@EXO evaluated by NTA. (C) Western blot analysis of CD9, CD63, CD81, TSG101, and calnexin. (D) Relaxation properties of ESIONPs@EXO. (E) T1 and T2 weighted MR images of ESIONPs@EXO at different concentrations (measured on a 3 T MR scanner). 1/T1 (F) and 1/T2 (G) relaxation rates of ESIONPs@EXO at different concentrations.

Next, ESIONPs@EXO were engineered using a pure biological method with BMMs (Figure 1A). ESIONPs were used to coincubate with BMMs for 24 h. Then the exosomes were isolated from the supernatant after another 24 h incubation. The characterization of exosomes derived from ESIONPs-treated macrophages were investigated. The result of TEM showed that both exosomes exhibited a cup-shaped structure (Figure 2A). Nanoparticle tracking analysis (NTA) showed that the exosomes of EXO and ESIONPs@EXO presented average sizes of 130.8 and 124.9 nm, respectively (Figure 2B). The contents of CD63, CD9, CD81, and TSG101 were detected in both exosomes, which were the markers of exosomes. Meanwhile, the expression of calnexin was not detected in exosomes, suggesting the high purity of isolated exosomes (Figure 2C). These results indicated that there was no physical difference between these two exosomes.

A recent study shows that extracellular vesicles can transport nanoparticles among different cells, indicating the possibility of cell-secreting nanoparticle-incorporated exosomes.^35^ Sparked by this report, we examined whether ESIONPs were incorporated into exosomes derived from ESIONPs-treated macrophages. Inductively coupled plasma mass spectrometry (ICP-MS) was used to measure the concentration of iron in the exosomes. The data indicated that the concentration of iron of exosomes from ESIONPs-treated macrophages was much higher than that from control macrophages (Figure S5). By normalization to the protein amount, 1 μg of exosomes from ESIONPs-treated macrophages contained approximately 54.37 ng of iron (Figure S5). The data of energy-dispersive X-ray spectroscopy (EDS) elemental mapping further confirmed the successful fabrication of ESIONPs@EXO (Figure S6). Furthermore, we assessed the MR phantom and relaxation properties of ESIONPs@EXO. The data showed that these exosomes exhibited good contrast effects and showed linear correlations between the 1/T1 and exosome concentration (Figure 2D–G). Being T1 contrast agents, similar properties of ESIONPs@EXO and ESIONPs were observed (Figure S7). These data suggested the success of engineering exosomes with ESIONPs by using a pure natural biological method.

### Ferroptosis-Inducing and Immuno-Modulatory Properties of ESIONPs@EXO

Being a new type of regulated cell death, ferroptosis is initiated by intracellular phospholipid peroxidation.^36,37^ Ferrous iron (Fe^2+^ and Fe^3+^) accumulation and lipid peroxidation play vital roles in the induction of ferroptosis.^37^ This process is under the precise control of glutathione peroxidase 4 (GPX4).^37^ Iron oxide nanoparticles are reported to induce ferroptosis of cancer cells and endothelial cells at relatively high concentrations.^38−40^ Hydroxyl radicals produced via the Fenton reaction catalyzed by iron-based nanomaterials are commonly ferroptosis activators.^41^ However, it is difficult for iron-based nanomaterials to accumulate in the lipid bilayer and to produce a hydroxyl radical there. Most iron-based nanomaterials localize in the cytoplasm, and the generated hydroxyl radical is prevented from initiating intrabilayer lipid peroxidation.^39^ Thus, high doses of iron-based nanomaterials are required to induce ferroptosis. Exosomes possess a lipid bilayer and membrane structure, which are easily fused to lipid bilayer in cells.^1^ Thus, ESIONPs@EXO were speculated to deliver ESIONPs to the lipid bilayer more efficiently.

**Figure 3:**
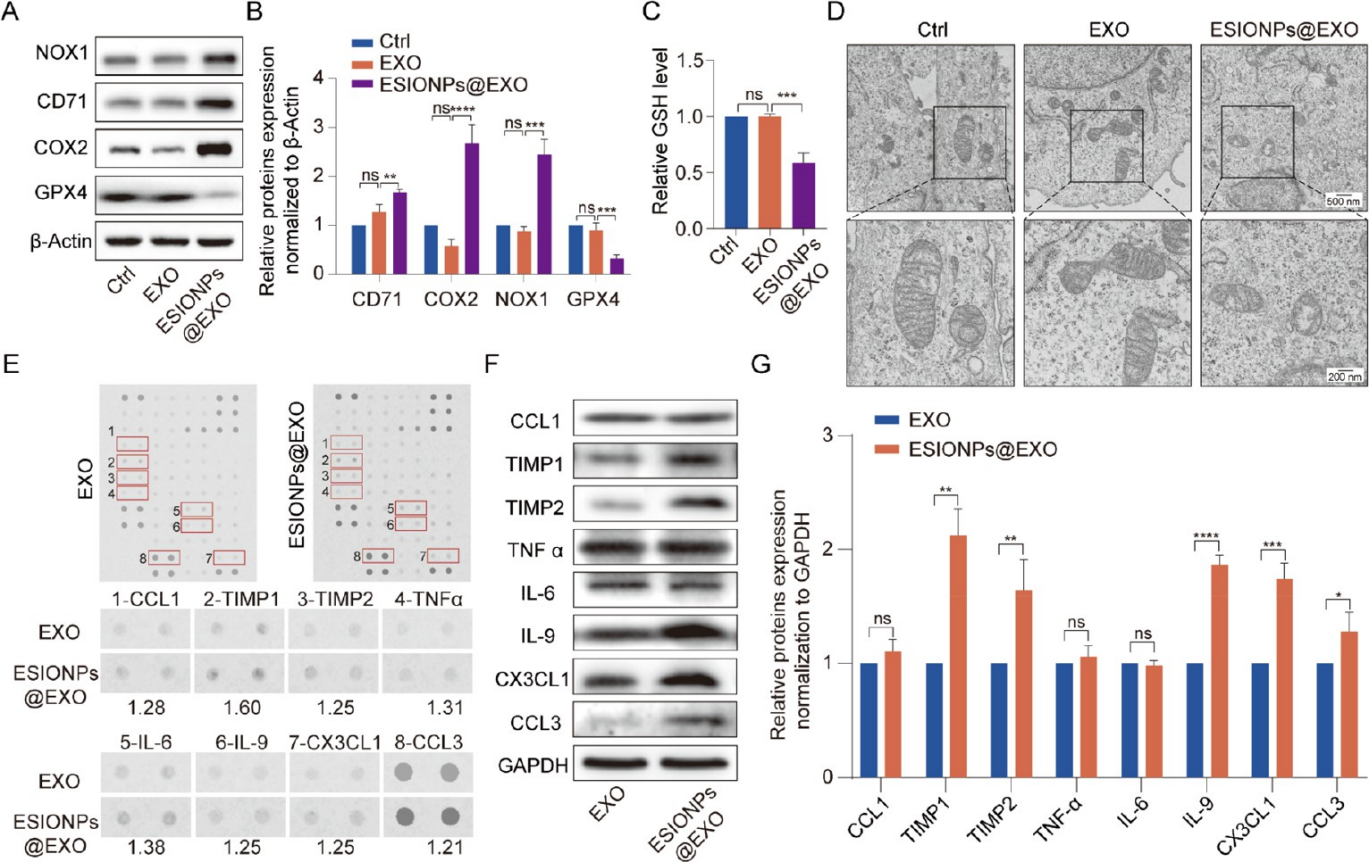
Ferroptosis-inducing and immuno-modulatory properties of ESIONPs@EXO. (A) The protein levels of NOX1, CD71, COX2, and GPX4 in ESIONPs@EXO-treated C166 determined by Western blot. (B) Statistical result of the protein levels in ESIONPs@EXO-treated C166. (C) Relative GSH level in ESIONPs@EXO-treated C166. (D) TEM representative images of mitochondria in C166 cells after treatment with ESIONPs@EXO for 24 h. Scale bar: 500 nm (upper) and 200 nm (lower). (E) Contents of various cytokines/chemokines in ESIONPs@EXO determined by protein array analysis. (F) The protein levels of CCL1, TIMP1, TIMP2, TNFα, IL-6, IL-9, CX3CL1, and CCL3 in EXO and ESIONPs@EXO determined by Western blot. (G) Statistical results of the protein levels in ESIONPs@EXO. Data were presented as means ± SD, *n* = 3, two-tailed *t* test, one-way ANOVA; *P < 0.05, **P < 0.01, ***P < 0.001, ****P < 0.0001.

Here, our data revealed that ESIONPs (250 μg/mL) did not reduce the viability and did not cause toxicity to vascular endothelial cells (C166) and melanoma cells (B16) (Figures S8 and S9), whereas ESIONPs@EXO (100 μg/mL) decreased the viability of C166 and B16 (Figure S10). Further data showed that ESIONPs@EXO suppressed the expression of GPX4, while it increased the expression of cyclooxygenase 2 (COX2), nicotinamide adenine dinucleotide phosphate oxidase 1 (NOX1), and CD71 in C166 (Figure 3A,B). Meanwhile, ESIONPs@EXO significantly decreased the cellular glutathione (GSH) level (Figure 3C). TEM images of C166 showed swelled mitochondrion, which was one of the hallmarks of ferroptosis (Figure 3D). Similar results were acquired in B16 cells, as ESIONPs@EXO decreased the levels of GPX4 and cellular GSH, while it increased the levels of COX2, NOX1, and CD71 significantly (Figure S11). However, ESIONPs did not induce ferroptosis of C166 and B16 with a concentration of 250 μg/mL (Figures S12 and S13). Interestingly, ESIONPs@EXO derived from BMMs could induce ferroptosis of C166 and B16 with a concentration of 100 μg/mL, suggesting that ESIONPs@EXO exhibited a better efficiency in ferroptosis induction than ESIONPs.

To confirm the specific induction of ferroptosis by ESIONPs@EXO, we further evaluated the effect of ESIONPs@EXO on necrosis, apoptosis, and autophagy. ESIONPs@EXO treatment did not increase the number of propidium iodide positive cells, indicating that ESIONPs@EXO did not cause necrosis of C166 and B16 cells (Figure S14A,B, Figure S15A,B). Terminal deoxynucleotidyl transferase (TdT) dUTP nick-end labeling (TUNEL) assay was used to assess the effect of ESIONPs@EXO on apoptosis, which showed that ESIONPs@EXO did not increase the number of TUNEL-positive cells (Figure S14C,D, Figure S15C,D). The expression of caspase 3, an apoptotic marker, in C166 and B16 did not change after treatment with ESIONPs@EXO (Figure S14E,F, Figure S15E,F). The results indicated that ESIONPs@EXO did not cause cell apoptosis. Furthermore, LC3 levels was used to determine the effect of ESIONPs@EXO on autophagy, which showed no difference among three groups (Figure S14G,H, Figure S15G,H). These data suggested that ESIONPs@EXO could specifically induce ferroptosis in C166 and B16 cells.

M1 macrophages exhibit good antitumor and antiangiogenesis functions.^15,42,43^ Our results showed that ESIONPs@EXO could induce M1 macrophage polarization in macrophages (Figure S16). A concentration of 250 μg/mL ESIONPs induced M1 polarization of macrophages (Figure S4), whereas ESIONPs@EXO could induce M1 polarization of macrophages at 100 μg/mL (about 5.4 μg/mL iron). The results suggested that the incorporation of exosomes with ESIONPs would largely improve the immunotherapeutic efficiency of ESIONPs, and the bioactive molecules inside exosomes might play a role. To clarify the functional molecules of ESIONPs@EXO, forty-eight cytokines were evaluated using mouse cytokine array panel. There were eight cytokines that were different between ESIONPs@EXO and control exosomes at the exosomal level: TNF-α, tissue inhibitor of metalloproteinases 1 (TIMP1), tissue inhibitor of metalloproteinases 2 (TIMP2), chemokine (C-X3-C motif) ligand 1 (CX3CL1), interleukin 9 (IL-9), interleukin 6 (IL-6), chemokine (C–C motif) ligand 3 (CCL3), and chemokine (C–C motif) ligand 1 (CCL1) (Figure 3E). The levels of these cytokines in ESIONPs@EXO were further confirmed using Western blot analysis. The data showed that the levels of TIMP1, TIMP2, CCL3, CX3CL1, and IL-9 were much higher in ESIONPs@EXO, whereas TNFα, IL-6, and CCL1 showed no differences between ESIONPs@EXO and control exosomes (Figure 3F,G).

Both TIMP1 and TIMP2 are natural inhibitors of the matrix metalloproteinases (MMPs), and the deficiency of TIMP1 and TIMP2 promotes angiogenic M2 macrophage polarization.^44,45^ CCL3 is reported to induce M1 macrophage polarization in necrotizing enterocolitis.^46^ IL-9 is a cytokine with potent proinflammatory properties and can stimulate antitumor M1 macrophages polarization in lung cancer.^47,48^ CX3CL1 is reported to promote M1 macrophage polarization in ankylosing spondylitis.^49^ These studies suggested that cytokines as TIMP1, TIMP2, CCL3, IL9, and CX3CL1 in ESIONPs@EXO promoted M1 macrophage polarization. Meanwhile, CX3CL1 deficiency is reported to suppress cell ferroptosis via increasing the levels of GSH and GPX4.^50^ The increased levels of CX3CL1 in ESIONPs@EXO contribute to ferroptosis of C166 and B16 cells. Together with the results above, ESIONPs@EXO exhibited excellent ferroptosis inducing and immuno-modulatory properties, which showed potential in pathological angiogenesis therapy.

### ESIONPs@EXO Inhibits angiogenesis *In Vitro*

**Figure 4:**
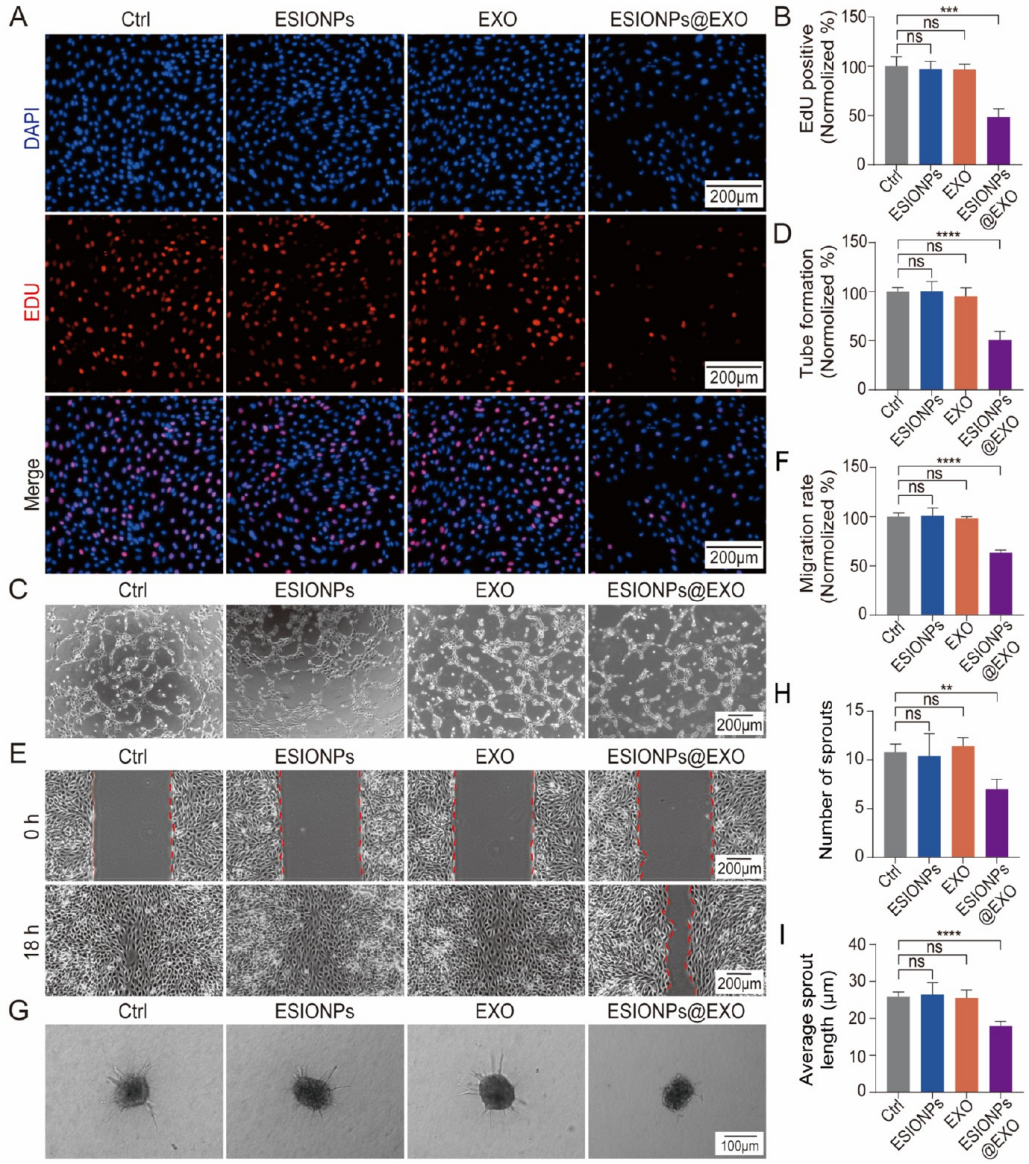
ESIONPs@EXO inhibit angiogenesis *in vitro*. Representative images (A) and quantification (B) of C166 in EdU incorporation assay after exposure to ESIONPs@EXO for 24 h. EdU-positive (red) and Hoechst-positive (blue) cells represent proliferating and total cells, respectively. Scale bar: 200 μm. Representative images (C) and quantification (D) of C166 for tube formation assay after treatment with ESIONPs@EXO for 24 h. Scale bar: 200 μm. Representative images (E) and quantification (F) of C166 for migration after exposure to ESIONPs@EXO for 24 h. Scale bar: 200 μm. Representative images (G) and quantification (H,I) of C166 for sprouting assay treated with ESIONPs@EXO for 24 h. Scale bar: 100 μm. Data were presented as means ± SD, *n* = 3, one-way ANOVA; **P < 0.01, ***P < 0.001, ****P < 0.0001.

To investigate the role of ESIONPs@EXO on angiogenesis, we conducted experiments using C166 cells. Immunofluorescent data indicated that EXO and ESIONPs@EXO were easily internalized by C166 (Figure S17). EdU stains the deoxyribonucleic acid (DNA) of proliferating cells directly, which is widely used for the assessment of cell proliferation. The data showed that ESIONPs@EXO suppressed the growth of C166 compared with Ctrl, ESIONPs and EXO groups (Figure 4A,B). Tube formation assay is widely used to assess angiogenic potential of ECs *in vitro*.^3^ Our results showed that ESIONPs@EXO suppressed the tube formation ability of C166 significantly (Figure 4C,D). Cell migration and sprouting are the hallmarks for vascular expansion.^3^ Wound healing assay was used to evaluate the effect of ESIONPs@EXO on cell migration. The results showed that ESIONPs@EXO apparently suppressed cell migration (Figure 4E,F). Furthermore, ESIONPs@EXO significantly suppressed endothelial cell sprouting, as it inhibited the sprout numbers and sprout length (Figure 4G–I). Next, the effect of ESIONPs@EXO on tumor cells (B16) was evaluated. The data showed that ESIONPs@EXO were internalized by B16 cells and inhibited B16 cell proliferation and migration significantly (Figure S18, Figure S19A–D). Besides, the effect of ESIONPs@EXO on cell invasion was assessed with transwell chamber. The data showed that ESIONPs@EXO inhibited B16 cell invasion significantly (Figure S19E,F). These *in vitro* data suggested that ESIONPs@EXO exhibited potential in the inhibition of angiogenesis.

### ESIONPs@EXO Targets Pathological Angiogenesis *In Vivo*

**Figure 5:**
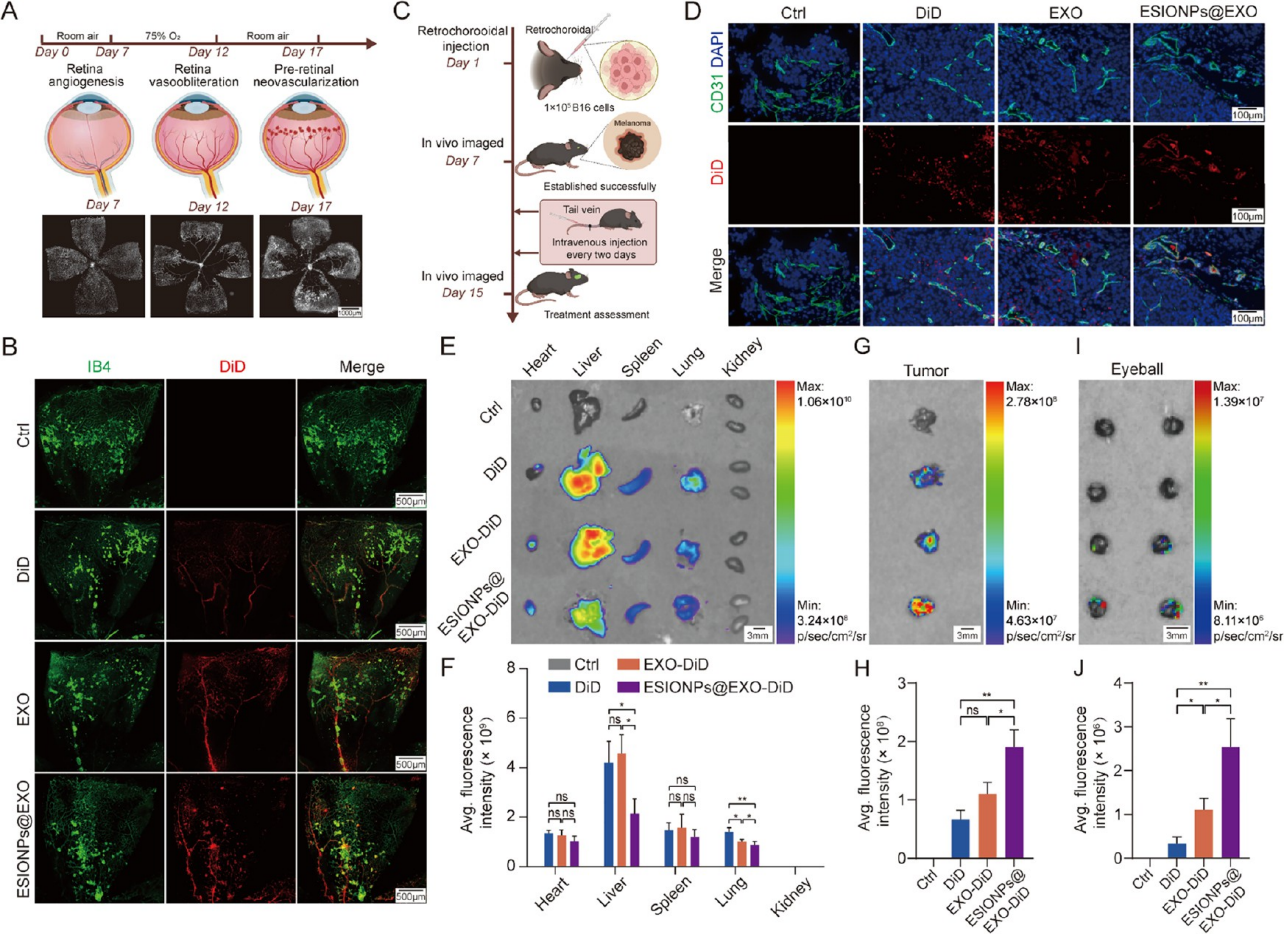
ESIONPs@EXO target pathological angiogenesis *in vivo*. (A) Schematic diagram of oxygen-induced retinopathy in mice. Scale bar: 1000 μm. Created with BioRender.com. (B) Fluorescent images of mouse retinal vascular stained with IB4 (green), DiD (red), DiD-EXO (red), and DiD-ESIONPs@EXO (red) in oxygen-induced retinopathy model. Scale bar: 500 μm. (C) Schematic diagram of the xenograft model showing tumor implantation and treatment time. Created with BioRender.com. (D) Fluorescent images of mouse melanoma tumor stained with DAPI (blue), CD31 (green), DiD (red), DiD-EXO (red), and DiD-ESIONPs@EXO (red) in xenograft model. Scale bar: 100 μm. (E) *Exvivo* images of organs and (F) average fluorescence intensity showing the organ distribution of DiD, DiD-EXO, and DiD-ESIONPs@EXO in tumor-bearing mice. Scale bar: 3 mm. (G) *Ex vivo* images of tumors and (H) average fluorescence intensity showing the tumor distribution of DiD, DiD-EXO, and DiD-ESIONPs@EXO in tumor-bearing mice. Scale bar: 3 mm. (I) *Ex vivo* images of eyeballs and (J) average fluorescence intensity showing the eyeball distribution of DiD, DiD-EXO and DiD-ESIONPs@EXO in OIR mice. Scale bar: 3 mm. Data were presented as means ± SD, *n* = 3 biological replicates, one-way ANOVA; *P < 0.05 and **P < 0.01.

Pathological angiogenesis occurs because of the imbalance of pro- and antiangiogenic signaling, and the abnormal vascular is characterized by dilated, tortuous, and hyperpermeable vessels.^51^ Different from physiological angiogenesis, pathological angiogenesis is usually hyperpermeable and nanosized substances are easily leaked through the vessel.^19^ These leaking vessels are exploited to design pathological angiogenesis targeting drugs. The enhanced permeability and retention (EPR) effect is an important concept for solid tumor targeting in nanomedicine, which is partially attributed to pathological angiogenesis.^52,53^ The oxygen-induced retinopathy (OIR) model is applied for the investigation of pathological retinal angiogenesis. As shown in Figure 5A, mice aged postnatal 7 days (P7) are bred in hyperoxia for 5 days. Then, the mice aged postnatal 12 days (P12) are brought to room air, leading to relatively low oxygen levels. The relative hypoxia results in pathological angiogenesis and avascular area in the retina and the retinopathy peaks on postnatal 17 day (P17). The data showed that compared with DiD alone, ESIONPs@EXO stained with DiD predominantly localized in the neovascular region (Figure 5B).

We then constructed an ocular melanoma model as previously described.^54^ As shown in Figure 5C, about 1 × 10^5^ B16 cells were injected into the mice choroid, and the tumor was assessed 7 days later. The pathological angiogenesis targeting ability of ESIONPs@EXO in ocular melanoma was evaluated. The data showed that ESIONPs@EXO mainly localized around blood vessels, whereas DiD and EXO groups did not show an apparent pattern (Figure 5D). To better demonstrate the pathological angiogenesis targeting ability of ESIONPs@EXO, we evaluated their distribution in live mice. The data revealed that the fluorescence intensity in the ESIONPs@EXO group was higher in ocular tumor and eyeball than the other groups (Figure 5E–J). Meanwhile, ESIONPs@EXO mainly accumulated in the liver and no signal was detected in the kidney, suggesting that they were metabolized and cleared through the liver. These data indicated that ESIONPs@EXO exert good pathological angiogenesis targeting activity. The nanoplatform was highly biocompatible and able to target leaky pathological angiogenesis through intravenous administration, which could largely avoid the side effects resulting from repeated intravitreal injections.

### ESIONPs@EXO Suppresses Pathological Retinal Angiogenesis

**Figure 6:**
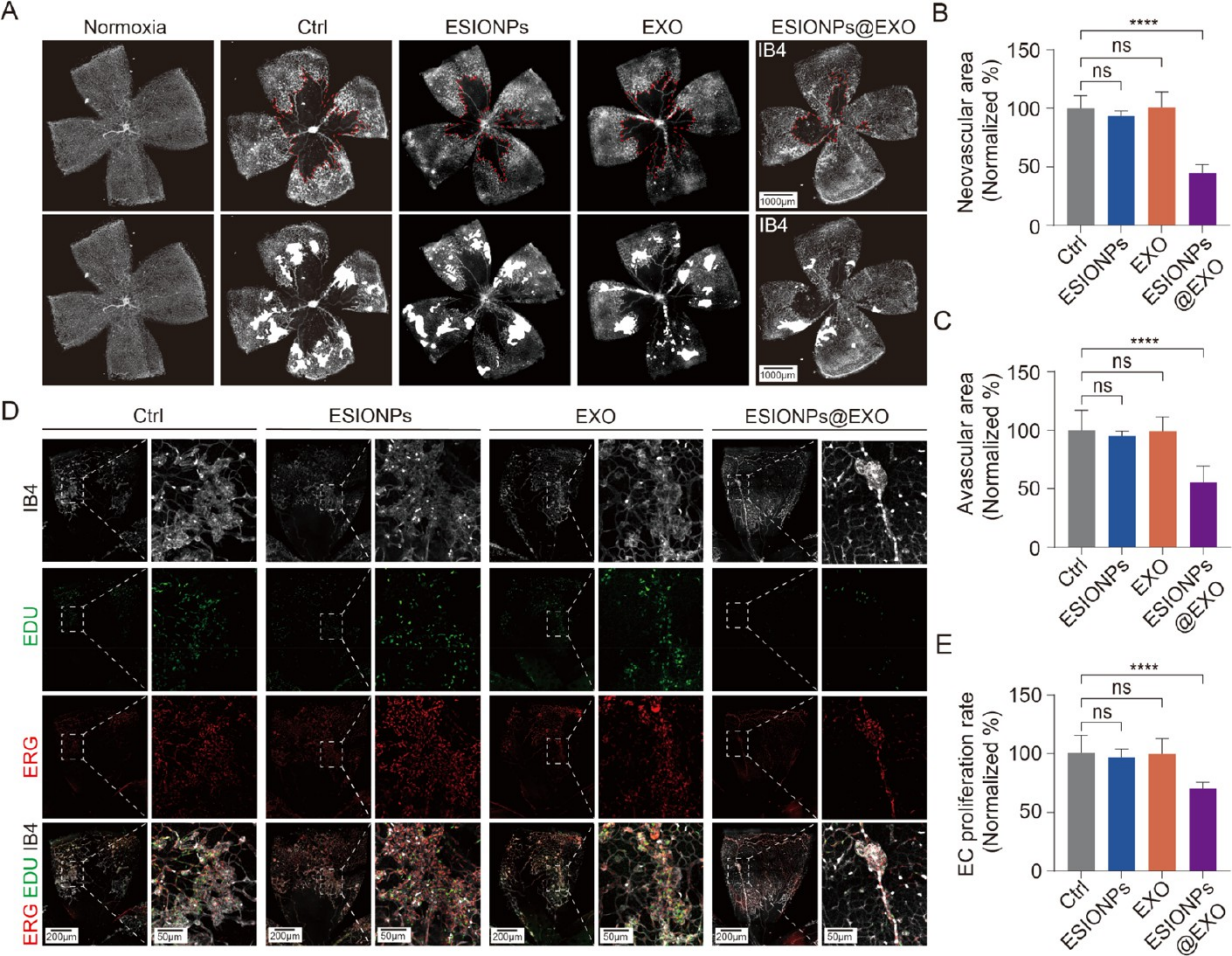
ESIONPs@EXO suppress pathological retinal angiogenesis. (A-C) The administration of ESIONPs@EXO into mice resulted in a significant amelioration of oxygen-induced retinopathy (A), as evidenced by a reduction in both neovascularization (B) and nonperfusion area (C). The red dotted line indicates avascular area in the central retina, and the white area represents the neovascular tufts in retina. Scale bar: 1000 μm. (D) Representative immunofluorescence image of retinas with P17 OIR mice. EdU (green), ERG (red), and IB4 (white) represent proliferating cells, endothelial cells, and blood vessel, respectively. Proliferating ECs are shown in yellow (EdU and ERG double-positive). Scale bar: 200 and 50 μm. (E) Statistical result of proliferating ECs. Data was presented as means ± SD, *n* = 8, one-way ANOVA; ****P < 0.001.

The OIR model is further used to evaluate the antiangiogenic effect of ESIONPs@EXO *in vivo*. When the mice were brought to room air (P12), the mice were randomly divided into four groups *via* tail vein injection: phosphate-buffered saline (PBS), ESIONPs, EXO, and ESIONPs@EXO. The results suggested that compared with PBS, ESIONPs, and EXO groups, ESIONPs@EXO significantly inhibited the pathological neovascularization (Figure 6A,B). Meanwhile, the avascular areas were decreased after the treatment of ESIONPs@EXO (Figure 6A,C).

The pathological neovascularization is characterized by uncontrollable vascular endothelial cell (EC) growth. Therefore, we assessed the effect of ESIONPs@EXO on EC proliferation. The result indicated that ESIONPs@EXO apparently inhibited the proliferation of ECs *in vivo* (Figure 6D,E). Furthermore, the toxicity of ESIONPs@EXO to the retina was assessed. The data indicated that ESIONPs@EXO did not lead to apparent damage to the retina (Figure S20). For the treatment of pathological retinal neovascularization, anti-VEGF reagents have been widely used in clinic.^19^ However, repeated intravitreal injection of anti-VEGF can lead to photoreceptor atrophy in some patients as the receptors of VEGF are detected in retinal neurons.^55,56^ Drug resistance or insufficient responses to anti-VEGF therapy is another challenge that needs to be resolved.^57^ ESIONPs@EXO exhibited good antiangiogenic roles through the VEGF-independent mechanism, which shows potential for the treatment of pathological retinal neovascularization.

### ESIONPs@EXO Inhibits Tumor Angiogenesis and Tumor Growth

**Figure 7:**
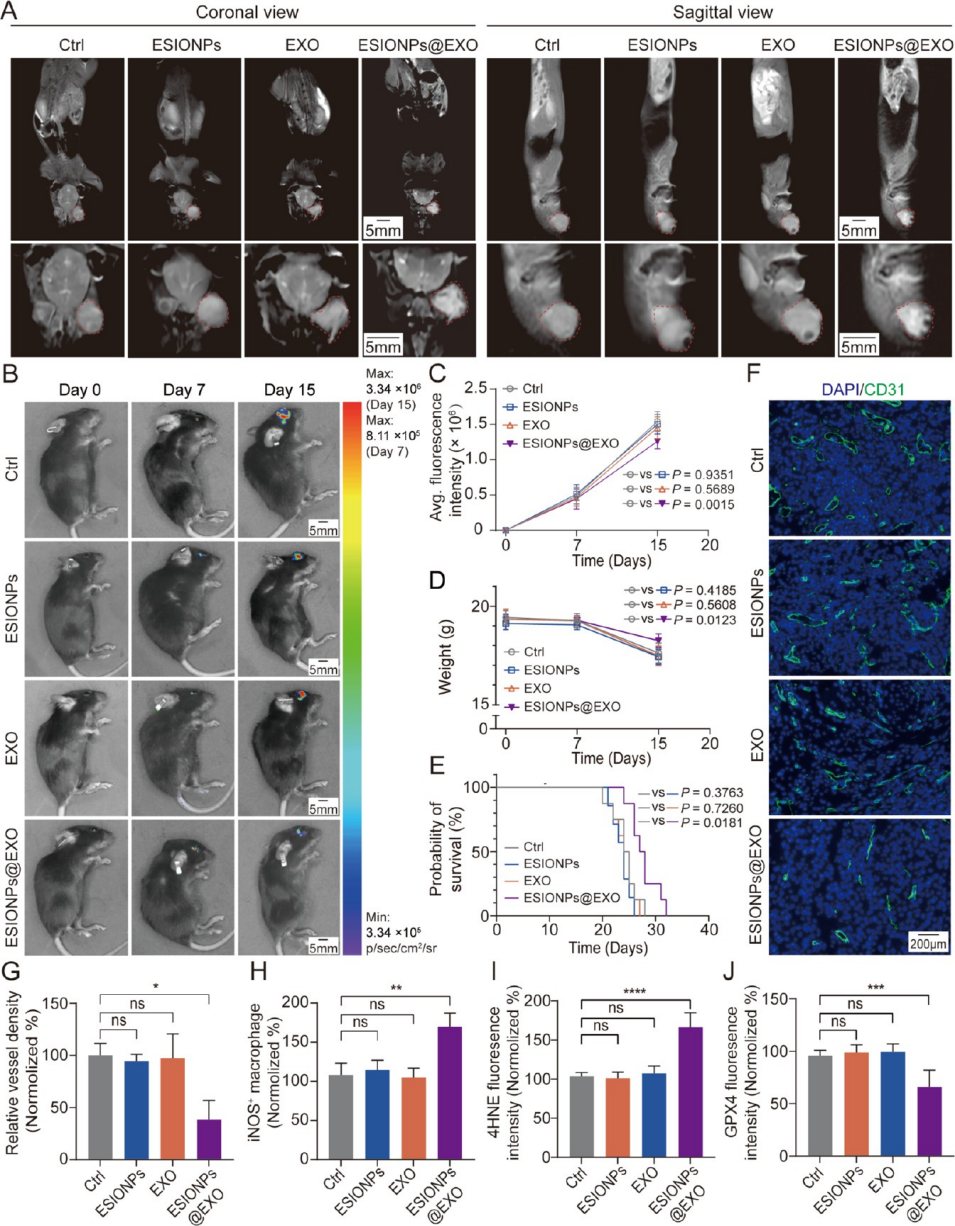
ESIONPs@EXO inhibit tumor angiogenesis and tumor growth. (A) ESIONPs@EXO enhance Coronal (left) and Sagittal (right) T1-weighted MRI of tumor-bearing mice *in vivo*. Red dot line indicateS the tumors. (B) Bioluminescence images and (C) average fluorescence intensity of luciferase-expressing B16-tumor-bearing C57BL/6J mice treated with ESIONPs@EXO. PBS (gray), ESIONPs (blue), EXO (orange), and ESIONPs@EXO (purple). **P = 0.0015 for ESIONPs@EXO vs PBS. (D) Average body weight of surviving mice in each group. PBS (gray), ESIONPs (blue), EXO (orange), and ESIONPs@EXO (purple). *P = 0.0123 for ESIONPs@EXO vs PBS. Data was presented as means ± SD, *n* = 8 biological replicates, one-way ANOVA; *P < 0.05 and **P < 0.01. (E) Kaplan–Meier survival curve of luciferase-expressing B16-tumor-bearing C57BL/6J mice in each group. PBS (gray), ESIONPs (blue), EXO (orange), and ESIONPs@EXO (purple). *P = 0.0181 for ESIONPs@EXO vs PBS. Log rank test was utilized for comparisons, *n* = 8 biological replicates, *P < 0.05. (F) Representative immunofluorescence images of mouse melanoma tumors stained with DAPI (blue) and CD31 (green). Scale bar: 200 μm. (G) Quantification of CD31 positive area in mice melanoma. (H) The ratio of iNOS + macrophage in tumor-bearing mice after treatment with ESIONPs@EXO. (I) Quantification of 4HNE fluorescent intensity in melanoma of mice. (J) Quantification of GPX4 fluorescent intensity in mice melanoma. Data was presented as means ± SD, *n* = 5 biological replicates, one-way ANOVA; *P < 0.05, **P < 0.01, ***P < 0.001, and ****P < 0.0001.

Our previous work indicates that ESIONPs exhibit excellent performance as T1MRI contrast agents.^26^ We found that ESIONPs@EXO maintained the properties of ESIONPs as a T1MRI contrast agent (Figure 2E). Furthermore, we evaluated their performance *in vivo*. The data showed that injection of ESIONPs@EXO significantly enhanced the signal in ocular melanoma compared to other groups (Figure 7A). The results of Prussian blue staining further confirmed that an increased iron element was detected in the tumor tissue after treatment with ESIONPs@EXO (Figure S21). The result indicated that ESIONPs@EXO penetrated through blood vessels and accumulated in the ocular melanoma, which also could be used for diagnosis.

Next, the inhibition of ocular melanoma growth and the effect on mice survival of ESIONPs@EXO were evaluated. After a 7-day period of tumor implantation, the mice were randomly divided into four groups: PBS, ESIONPs, EXO, and ESIONPs@EXO. The growth of tumor was monitored using bioluminescence imaging every 7 days. The results indicated that ESIONPs@EXO treatment suppressed tumor growth significantly (Figure 7B,C). Furthermore, the results showed that ESIONPs@EXO attenuated the weight loss in tumor-bearing mice (Figure 7D). Meanwhile, our data showed that ESIONPs@EXO apparently prolonged the survival of tumor-bearing mice (Figure 7E). Subsequently, immunostaining was employed to detect *K*_i_-67 in tumor tissue to assess its therapeutic impact *in vivo*. The findings revealed a reduction of *K*_i_-67 positive cells in tumor tissue upon treatment with ESIONPs@EXO (Figure S22).

Blood vessels are essential for solid tumors to grow and metastasize, and the disruption of which has been shown to be promising therapeutic option.^58,59^ Our results revealed that compared with PBS, ESIONPs, and EXO, ESIONPs@EXO treatment significantly suppressed tumor angiogenesis (Figure 7F,G). Immunotherapy and ferroptosis have shown great potential in cancer therapy.^60−62^ The results indicated that ESIONPs@EXO treatment increased the ratio of M1 macrophages in tumor tissue (Figure 7H, Figure S23). Meanwhile, ESIONPs@EXO increased 4-hydroxynonenal (4-HNE) levels and decreased GPX4 levels in ocular melanoma (Figure 7I,J, Figure S24). These data indicated that ESIONPs@EXO suppressed pathological angiogenesis and exhibited ferroptosis-inducing and immuno-modulatory properties *in vivo*.

Furthermore, the systematic toxicity of ESIONPs@EXO was evaluated. The morphology of major organs (lung, liver, kidney, heart, and spleen) was stained with H&E. The morphology did not exhibit apparent abnormality among different groups, indicating that ESIONPs@EXO did not cause organ toxicity (Figure S25). Blood biochemistry tests and routine examinations were further studied to confirm the safety of ESIONPs@EXO. The results showed that ESIONPs@EXO did not cause abnormal changes in these examinations, demonstrating negligible systemic toxicity of ESIONPs@EXO (Figure S26, Figure S27).

## Conclusions

In summary, this study presents a simple biological method to incorporate ESIONPs into exosomes, and ESIONPs@EXO exhibit multiple functions as magnetic imaging, ferroptosis inducing, and immunotherapy. ESIONPs@EXO target pathological angiogenesis in angiogenic retinopathy and uveal melanoma and suppress angiogenesis through a VEGF-independent mechanism. Therefore, it can provide an efficient strategy for the treatment of pathological angiogenesis with the potential for clinical translation.

## Experimental Methods

### Mice

Male C57BL/6J mice were obtained from Shanghai Jihui Experimental Animal Feeding Co., Ltd. (Shanghai, China). Mice were maintained under pathogen-free conditions. Mice were fed standard laboratory chow and kept on 12 h light/dark cycles. All operations were performed under sodium pentobarbital anesthesia, and effort was made to minimize pain. All animal experiments were performed in accordance with the NIH Guide for the Care and Use of Laboratory Animals and were approved by the Institutional Animal Care and Use Committee of Shanghai Changhai Hospital (CHEC (A.E) 2022-020).

### Isolation of BMMs

Isolation of bone marrow-derived macrophage (BMMs) was performed as described previously.^63^ Briefly, C57BL/6J mice aged 4–6 weeks were euthanized and soaked in 75% ethanol. Then, the femurs and tibias were removed completely with sterile forceps and scissors. Both ends of the bones were removed, and bone marrow cells were flushed out using 10 mL syringe with a 23-guage needle with ice cold DMEM. Then, we resuspended the obtained bone marrow with a 1 mL pipet tip. Next, a 40 μm sterile filter (Falcon brand, BD Biosciences) was placed on a 50 mL tube to filter the bone marrow suspension. The cell suspension was then transferred to 10 cm dish and cultured at 37 °C and 5% CO_2_. After 12 h, the supernatant was collected and centrifuged for 5 min at 600*g*. The isolated cells were resuspended in complete medium with 25 ng/μL recombinant mouse M-CSF (Peprotech, NJ, USA) and cultured for 6 days to form proliferative nonactivated cells.

### Preparation of ESIONPs@EXO

Fetal bovine serum (FBS) was ultracentrifuged at 100000*g* for 4 h to remove exosomes in bovine serum, named exosome-depleted FBS. To obtain ESIONP-containing exosomes (ESIONPs@EXO), macrophages were incubated in DMEM supplemented with 10% exosome-depleted FBS and 1% penicillin-streptomycin (exosome-depleted DMEM). After 24 h of treatment with ESIONPs (250 μg/mL), the supernatant was discarded, and cells were washed twice with PBS. Then, fresh exosome-depleted medium was supplemented and incubated with cells for another 24 h. Macrophage-derived exosomes were isolated by multistep centrifugation.^3^ Briefly, the harvested medium was centrifuged at 300*g* for 5 min to remove cells, 2000*g* for 20 min, and 10000*g* for 30 min to remove cell debris. Afterward, the supernatants were filtered through a filter (0.22 μm). The preprocessed supernatant was then ultracentrifuged at 150000*g* for 2 h at 4 °C using a Type 70 Ti rotor in a L-90 K ultracentrifuge (Beckman Coulter, USA). Then the exosome pellet was resuspended in phosphate-buffered saline (PBS) and used immediately or stored at −80 °C until use.

### Oxygen-Induced Retinopathy

The OIR mice model was used to observe the therapeutic effect of ESIONPs@EXO on neovascularization *in vivo*, which resembles human retinopathy of prematurity (ROP) and certain aspects of human proliferative diabetic retinopathy (PDR). OIR was induced by exposing C57BL/6J pups with mother to high oxygen (75 ± 0.5%) from P7–P12. Oxygen was continuously monitored by using an oxygen analyzer (XBS-03S, Hangzhou Aipu Instruments, Hangzhou, China). On P12, pups were placed to normoxia, randomly divided into four groups, and given intravenous injections of PBS (100 μL), ESIONPs (15.5 μg dispersed in 100 μL of PBS), EXO (200 μg dispersed in 100 μL of PBS), or ESIONPs@EXO (200 μg dispersed in 100 μL of PBS) every 2 days. The amount of ESIONPs was equal to that of ESIONPs@EXO normalized to the content of Fe. At P17, pups were euthanized, and their eyes were enucleated and fixed in 4% PFA for further immunofluorescent assays.

### *In Vivo* MRI of Mouse

The *in vivo* MRI of mice was tested using a clinical 3 T MR scanner (Siemens). On day 15 after the establishment of the xenograft model, mice were subjected to anesthesia through intraperitoneal injection of pentobarbital sodium (60 mg/kg). Then the mice were administered 100 μL of PBS, ESIONPs (15.5 μg dispersed in 100 μL of PBS), EXO (200 μg dispersed in 100 μL of PBS), and ESIONPs@EXO (200 μg dispersed in 100 μL of PBS) through the tail vein. The amount of ESIONPs was equal to that of ESIONPs@EXO normalized to the content of Fe. Following a 1 h interval, the mice were immobilized at the center of the MRI scanner coil, and MR imaging was conducted utilizing the following imaging sequence: TE = 33.14 ms, TR = 2000 ms, FOV = 40 × 40 cm^2^, matrix = 384 × 384, slice thickness = 3.0 mm.

### Statistical Analysis

Statistical analysis was performed with GraphPad Prism (GraphPad software 9.0, MD, USA). The measurement of two groups was analyzed by unpaired Student’s *t* test, and the comparison between multiple groups was performed by one-way analysis of variance (ANOVA). Survival analysis was performed by using Kaplan–Meier survival analysis. Each experiment was performed in triplicate, and *P* values <0.05 were considered as statistically significant (*P < 0.05; **P < 0.01; ***P < 0.001; ****P < 0.0001).

## Data Availability

The data that support the findings of this study are available from the corresponding author upon reasonable request.

## Supporting Information Available

Detailed materials and methods, supplementary figures, and supplementary tables (PDF)
