# Supplementary material for: Iron Oxide Nanoparticles Engineered Macrophage-Derived Exosomes for Targeted Pathological Angiogenesis Therapy
Source: ACS Nano. 2024 Feb 27;18(10):7644–55. doi: 10.1021/acsnano.4c00699 (PMC10938920; doi:10.1021/acsnano.4c00699)
Supplement: Supplementary file 1 — nn4c00699_si_001.pdf [file nn4c00699_si_001.pdf]

Supporting Information

Iron oxide nanoparticles engineered macrophage-derived exosomes for targeted pathological angiogenesis therapy

Haorui Zhang[#, †], *Yu Mao*[‡, †], *Zheng Nie*[#, †], *Qing Li*[#], *Mengzhu Wang*[#], *Chang Cai*[#], *Weiju Hao*[§], *Xi Shen*[‖], *Ning Gu*[‡, *], *Wei Shen*[#, *], *Hongyuan Song*[#, *]

[#] Department of Ophthalmology, Shanghai Changhai Hospital, Shanghai, 200433, P.R. China

[‡] Nanjing Key Laboratory for Cardiovascular Information and Health Engineering Medicine, Institute of Clinical Medicine, Nanjing Drum Tower Hospital, Medical School, Nanjing University, Nanjing, 210093, P.R. China

[§] University of Shanghai for Science and Technology, Shanghai 200093, PR China

[‖] Department of Ophthalmology, Ruijin Hospital, Shanghai Jiao Tong University School of Medicine, Shanghai 200020, P.R. China.

[*]Correspondence should be addressed to Hongyuan Song (hongyuansong@hotmail.com); Wei Shen (shenwei@smmu.edu.cn); Ning Gu (guning@nju.edu.cn).

[†]Authors contributed equally to this work.

Experimental Section

Materials. ESIONPs were developed by Professor Ning Gu's Lab of Nanjing University, Nanjing, China.[1] 4′,6-diamidino-2-phenylindole (DAPI, 62247) were obtained from Thermo Fisher Scientific (MA, USA). Counting Kit8 (CCK8, TM772) was obtained from Dojindo Laboratories, (Dojindo, Japan). Cell-Light EdU Apollo567 In Vitro Kit (C10310-1) was purchased from RIBOBIO (Guangzhou, China). Ceturegel^{TM} Matrix High Concentration (40187ES08), (1,1'-Dioctadecyl-3,3,3',3'-Tetramethylindodicarbocyanine Perchlorate (DiD, 40758ES25) and D-Luciferin firefly potassium salt (40902ES02) were purchased from Yeasen Biotechnology (Shanghai, China). Triton-X-100 (P0096), BCA Protein Assay Kit (P0012), One-step TUNEL cell apoptosis detection kit (C1090) and GSH and GSSG Assay Kit (S0053) were obtained from Beyotime Biotechnology (Jiangsu, China). Fluorescein-conjugated isolectin-I B4 (FL-1201) was purchased from Vector Laboratories (Burlingame, USA). Calcein-AM/PI Live Cell/Dead Cell Double Staining Kit (CA1630) and Prussian Blue Iron Stain Kit (G1422) was purchased from Solarbio (Beijing, China). Antibodies to β-Actin (66009), Calnexin (10427), CD31 (11265), LC3 (14600), GAPDH (10494), TSG101 (28283), TNF-a (60291), IL-6 (21865), GPX4 (67763), Ki-67 (27309), COX2 (12375), NOX1 (17772) and CD71 (10084) were purchased from Proteintech Group (Wuhan, China). Antibodies to Caspase 3 (AF6311), CCL1 (DF9910), CD9 (AF5139), CD63 (AF5117) and CD81 (DF2306) were purchased from Affinity Biosciences (Jiangsu, China). Antibodies to CCL3 (A7568), CX3CL1 (A14198), IL-9 (A6630), TIMP1 (A1389) and TIMP2 (A20766) were purchased from ABclonal (Wuhan, China). Antibodies to 4HNE (bs6313R) was purchased from Bioss (Beijing, China). Antibodies to F4/80 (GB11027) was obtained from ServiceBio (Wuhan, China). Antibodies to iNOS (MAB9502)

was purchased from R&D Systems (Minneapolis, MN, USA). All of the cell culture plates and dishes were purchased from Corning Life Sciences (Bedford, MA, USA).

Cell culture. C166 cells were obtained from WHELAB cell company (Shanghai, China); B16 mouse melanoma cells were purchased from Procell Biotech Co. Ltd (Wuhan, China); RAW 264.7 cells were purchased from National Collection of Authenticated Cell Cultures (Shanghai, China). C166, B16 cells, Bone marrow-derived macrophages (BMMs) and RAW 264.7 cells were cultured in Dulbecco's Modified Eagle Medium (DMEM), which was purchased from Gibco (CA, USA). All growth media were supplemented with 10% fetal bovine serum (FBS) and 1% penicillin-streptomycin (Gibco, CA, USA). Cells were cultured at 37 °C and 5% CO_2. All cell lines were tested negative for mycoplasma.

CCK8 Assay. The CCK8 assay was performed using a CCK8 kit following the manufacturer's protocol. Briefly, cells were plated into 96-well plates (2×10^3 cells per well) in 100 μL culture medium for 24 h at 37°C. Then, cells were treated with PBS, ESIONPs, EXO or ESIONPs@EXO for 24 h. After that, CCK8 solution (100 μL/well) was added and cultured for another 2 h. Finally, the optical density (OD) was measured at 450 nm with a microplate reader (BioTek Synergy HT, USA).

Assessment of macrophage polarization. In order to assess the potential polarization effects of ESIONPs or ESIONPs@EXO on macrophages, BMMs and RAW264.7 macrophage cells (3×10^5 cells/mL) were cultured in 6-well plates overnight. Subsequently, macrophages were co-cultured with ESIONPs of various concentrations or ESIONPs@EXO for a duration of 24 hours. To determine the expression of M1 and M2 associated genes *in vitro*, macrophages were collected and RNA extraction was performed using the RNAsimple Total RNA Kit (Tiangen,

Shanghai, China). cDNA was obtained using Hifair® III 1st Strand cDNA Synthesis SuperMix (11141ES60, Yeason, Shanghai, China) according to the manufacturer's manual. Real-time quantitative polymerase chain reactions (qRT-PCR) were performed using Hieff UNICON® Power qPCR SYBR Green Master Mix (11184ES03, Yeason, Shanghai, China) with LineGene 9600 Plus detection system (Bioer Technology, Zhejiang, China) for amplification. PCR reactions consisted of 10 µL SYBR Green master mix, 2 µL experimental DNA sample, 0.4 µL of 10 µM forward and reverse primers (0.2 µM reaction concentration), and 7.2 µL nuclease-free water for a final reaction volume of 20 µL. The standard reactions were run with the following conditions: 95 °C for 2 min, 40 cycles of 95 °C denaturation for 10 s and 60 °C annealing and extension for 30s. The sequences of gene-specific primers were provided in Table S1. The relative expressions of specific genes were quantitated with the 2-ΔΔCt (cycle threshold) calculation method, using GAPDH as the reference housekeeping gene for the expression analyses. All samples were run in triplicate and averaged to define biological replicates. Experiments were repeated at least 3 times on different days.

Characterization of ESIONPs@EXO. Exosome protein concentration was quantified using the BCA protein assay kit. The morphology of the ESIONPs and exosomes was observed by Transmission electron microscopy (TEM; HT7800; Hitachi, Tokyo, Japan). To further confirm the presence of ESIONPs, Energy-Dispersive X-ray Spectroscopy (EDS) elemental mapping was performed. Exosome size and distribution were determined by nanoparticle tracking analysis (NTA) using an Electrophoresis & Brownian Motion Video Analysis Laser Scattering Microscopy (Zataview, Particle Metrix, Germany). The iron content present in the EXO and ESIONPs@EXO was quantitatively determined using ICP-MS (Agilent Technologies, 7800 ICP-MS). In order to assess the *in vitro* cellular uptake of ESIONPs@EXO, DiD-labelled EXOs

were employed to visualize the endocytosis of exosomes by C166 and B16 cells. Briefly, EXO (100 μg/mL) were incubated with DiD (5 μM/L) at 37 °C for 30 min, the rest of unbound DiD was then removed by centrifugation at 100 000g for 2 h at 4 °C. To visualize exosome internalization by C166 and B16, DiD-labelled EXOs were co-incubated with C166 and B16 for 8 h and analyzed using confocal microscopy Leica TCS SP5-II (Leica, Wetzlar, Germany).

Cell proliferation assay. Cellular proliferation was measured with Cell-Light EdU Apollo567 In Vitro Kit. Briefly, cells were seeded in 96-well plates at a density of 2×10^3 cells per well and treated with PBS, ESIONPs, EXO and ESIONPs@EXO for 24 h. Cells were co-incubated with 5-ethynyl-2-deoxyuridine (EdU) working solution (1:1000) for 2 hours at 37°C and then fixed with 4% paraformaldehyde for 30 minutes according to the manufacturer's protocol. Cells were then washed with glycine for 5 minutes, followed by two washes with 100 µL of 0.3% Trion X-100. Subsequently, the cells were incubated with 100 µL of Apollo Fluorescent Azide for 30 minutes at room temperature in the dark, followed by 3 washes with 100 µL of 0.3% Trion X-100. DAPI solution was incubated with sample for an additional 30 minutes, and the cells were washed 3 times with PBS. The proliferation was calculated as the number of EdU positive cells/the number of DAPI-stained cells. Images were acquired using a Biotek Cytation5 Imager and cells were counted using ImageJ software.

Cell invasion assay. Cell invasion was evaluated using a 24-well polycarbonate membrane insert (8-µm pore size, Corning) in accordance with the manufacturer's instructions. Briefly, the membrane was coated with Ceturegel™ Matrix (1 mg/mL, 50 μL; Yeasen, Shanghai, China) at 37 °C overnight. A total of 5×10^5 cells in serum-free medium were seeded in the upper compartment of invasion chambers. The lower compartment of the invasion chambers was filled

with culture medium containing 20% FBS. After 48 hours of incubation, the cells on the upper surface of the membrane were removed with a cotton bud. The migrated cells were fixed in 4% paraformaldehyde for 10 min and stained with 0.5% crystal violet in 2% ethanol for 5min at room temperature. The number of migrated cells were counted from five randomly selected visual fields under an inverted Olympus IX-81 microscope (Olympus, Tokyo, Japan).

Wound healing migration assay. The wound healing assay was performed to evaluate the ability of cell migration. The ibidi Culture-Insert, featured by two cell culture regions separated by a 500 μm wall, was employed to create the gap. The ibidi insert would ensure reliable results due to the precise dimensions of the cell-free region. Briefly, about $2×10^4$ cells were seeded in 2-well ibidi chamber plate to form a confluent monolayer. The cells were pretreated with PBS, ESIONPs, EXO and ESIONPs@EXO at 37 °C for 24 h, respectively. Then, the insert was pulled up and washed three times with PBS to remove the suspended cells. Next, cells were cultured with fresh medium with PBS, ESIONPs, EXO and ESIONPs@EXO. Images of the entire well were obtained at 0 h and 18 h using an Olympus IX-81 microscope (Olympus, Tokyo, Japan). All images captured from each experiment were processed using ImageJ software.

Tube formation assay. In order to assess the inhibitory effects of ESIONPs@EXO on angiogenic properties of C166 cells, a tube formation assay was conducted using a 15-well angiogenesis slide (Ibidi, Martinsried, Germany). Initially, 10 µL of Matrigel (12 mg/ml) was dispensed onto a pre-cooled well of the angiogenesis slide and allowed to sit for 1 hour at 4 °C to achieve a smooth consistency. The cells were pretreated with PBS, ESIONPs, EXO and ESIONPs@EXO at 37 °C for 24 h, respectively. Subsequently, the slide was solidified at 37°C for 30 minutes, followed by seeding of $1 × 10^4$ cells in 50 µL of DMEM per well onto the

aforementioned angiogenesis slide. Following a 3-hour incubation at 37°C, images were captured using an Olympus IX-81 microscope, and the length of the formed tubes was quantified using ImageJ software.

Sprouting assay. Spheroid sprouting assay was employed to assess the effects of ESIONPs@EXO on angiogenesis *in vitro*. Briefly, C166 cells were detached from the cell culture plate and were resuspended into medium containing PBS, ESIONPs, EXO or ESIONPs@EXO at a density of 2×10^4 cells/mL. The cells were combined with 1 mL of methocel stock solution and subsequently transferred to a sterile multichannel pipette reservoir. A volume of 25 μL of the solution was carefully dispensed onto a 10 cm square petri dish using a pipette. The dish was then inverted and placed in a cell culture incubator for a duration of 24 hours. Next, the spheroids were gently washed with 10 mL of PBS and transferred into a 15 mL conical tube. Spheroids were centrifuged at 200 g for 5 min and then resuspended in 2 mL of methanol containing 20% FBS. A total of 4 mL of collagen solution was combined with 0.5 mL of 10 x Medium 199, and the pH was adjusted by the addition of sterile ice-cold 0.2 N NaOH. Subsequently, 2 mL of the collagen/Medium 199 solution was mixed with a methanol solution containing spheroids. Following this, 1 mL of the spheroid-collagen solution was added to each well of a 24-well plate, which was then incubated at 37 °C for 30 minutes to allow for collagen polymerization. The spheroids were then stimulated with 200 μL of medium containing PBS, ESIONPs, EXO or ESIONPs@EXO for a duration of 24 hours in a humidified incubator. Images were captured using a microscope (IX81, Olympus), and the number of sprouts or vascular length were calculated using ImageJ software.

Dead-Live staining. Dead-Live cell staining was performed to assess the cytotoxicity *in vitro* using a live/dead staining kit. Briefly, cells were seeded at a density of 1×10^4 cells/well in 24-well plates and cultured for 12 h. Then, cells were co-incubated with PBS, ESIONPs, EXO and ESIONPs@EXO for 24 h. After that, cells were stained with calcein-AM (living cells) and propidium iodide (necrotic cells) at 37 °C for 30 min in the dark. Images of cellular fluorescence were obtained using a fluorescence Olympus IX-81 microscope, and the number of Dead and Live cell were calculated using ImageJ software.

Cytokine Array. To detect inflammatory cytokines in the ESIONPs@EXO, we applied the mouse Inflammation Array 1 Kit (AAM-INF-G1; Raybiotech,Inc., Norcross, GA, USA) and each sample was performed twice. The analysis was performed strictly according to manusfacturer's instructions. The obtained EXO samples were resuspended in PBS and the protein content was determined using the BCA assay kit. The array was dried at room temperature for 2 hours, and then 500 μg/mL lysate was added to on the array and incubated overnight at 4°C. After rinsing the array, we added 100 μL of secondary antibody solution and incubated for 2 hours at room temperature. The array was washed twice. Then, 100 μL of detection solution was added to the array and incubated for 1 hour at room temperature. Finally, the microarrays were scanned, and signals were extracted using an InnoScan 310 microarray scanner (Innopsys, Carbonne, Midi-Pyrénées, France). The fluorescence data were converted to concentration values using RayBio analysis software (Raybiotech, Inc., Norcross, GA, USA).

GSH Measurement. The intracellular concentration of total GSH was measured using a GSH and GSSG Assay Kit according to the manufacturer's protocol. The cells were pretreated with

PBS, ESIONPs, EXO and ESIONPs@EXO at 37 °C for 24 h, and cells were harvested and washed with PBS. Then, protein removal reagent M (30 μL) was added to each cell pellet (10 mg) and vortexed. The samples were then subjected to two cycles of freeze-thaw between liquid nitrogen and 37 °C water bath. The mixture was left at 4°C for 5 minutes, then centrifuged at 10,000 g for 5 minutes at 4 °C to remove cellular debris. Then, the supernatant was used for total GSH measurements. Next, 10 μL sample extracts and 150 μL GSH assay liquid were mixed. After incubation for 5 minutes at 25 °C, the reaction was started by addition of 50 μL NADPH (0.5 mg/mL). The absorbance at a wavelength of 412 nm was determined using a BioTek Cytation5 plate reader (Biotek, Winooski, USA).

Western Blotting. Total protein was extracted from cells or exosomes using RIPA lysis buffer (Beyotime, Shanghai, China) containing 2% protease and protease inhibitor. Protein concentrations were determined using a standard bisphenolic acid assay (BCA, Beyotime, Shanghai, China) according to the manufacturer's instructions. Samples were prepared in 5 x SDS-PAGE loading buffer and loaded onto the gel after heating at 95°C for 10 minutes. Protein samples and the protein markers (20 μL samples per lane) were separated by 12.5% sodium dodecyl sulfate-polyacrylamide gel electrophoresis (SDS-PAGE) at 120 volts for 50 min, and then transferred to PVDF membranes at 100 V for 110 minutes. Membranes were blocked with 5% nonfat dry milk in TBST (Tris-buffered saline with 0.1% Tween-20) for 2h at room temperature (RT). The membrane was then briefly rinsed in TBST and incubated with the following primary antibodies diluted in 5% BSA in TBST overnight at 4°C: (Primary antibodies used this study were summarized in Table S2). After being washed four times in TBST, the membrane was incubated in horseradish peroxidase-conjugated secondary IgG antibody (in 5% non-fat dry milk) for 1.5 h at RT. After another four washes in TBST, chemiluminescence was

detected with ECL Western blotting detection reagent (Merck-Millipore, Darmstadt, Germany). Images were captured using Gelview 6000Plus Image Capture System (Guangzhou Biolight Biotechnology Co., Ltd, Guangzhou, China) and quantified using ImageJ software.

Retina Immunofluorescence. Immunofluorescence (IF) staining of whole-mounted retinas and retinal sections were performed to assess angiogenesis *in vivo* as previously described.[2] Briefly, eyes were enucleated and fixed in 4% paraformaldehyde (PFA) for 30 min at RT. Then retinas were completely isolated under the microscope and blocked with 5% BSA in PBS containing 0.3% Triton-X-100 for 30 min at RT. After three washes with PBS, retinas were incubated with FITC-conjugated Isolectin B4 (IB4) and ERG primary antibodies (diluted 1:250 in 5% BSA and 0.3% Triton-X-100 in PBS) overnight at 4 °C. After that, retinas were washed 3 times for 5 minutes each time in PBS and incubated with secondary antibodies (diluted 1:250 in 5% BSA and 0.3% Triton-X-100 in PBS) for 2 h at RT. In the final step, the retinas were washed three times with PBS, and were mounted on glass slides and sealed with a fluorescent mounting medium. Slides were imaged with a confocal microscopy Leica TCS SP5-II (Leica, Wetzlar, Germany) and Image J was used to quantify the avascular and neovascular areas.

***In vivo* xenograft mouse model and fluorescent imaging.** To evaluate the therapeutic effect of ESIONPs@EXO on tumor growth *in vivo*, B16 melanoma cell with a luciferase-encoding lentivirus was used to establish uvea melanoma xenograft model. The lentivirus vector is GV260: Ubi-MCS-firefly-Luciferase-IRES-Puromycin (Shanghai Jikai Gene Chemical Technology Co., Ltd., Shanghai, China). Male C57BL/6 mice (4–6 weeks) were anesthetized and 1×10^5 B16 cells in 2 μL serum-free DMEM were injected into right choroid per mouse. The IVIS Spectrum Live Imaging System (PerkinElmer, Branford, USA) was utilized to perform

luminescent imaging 7 days post-implantation. D-Luciferin firefly potassium salt was administered intraperitoneally (150 mg/kg) to mice, and the mice were subsequently randomized into four groups (n = 8 per group). The mice were then intravenously injected with PBS (100 μL), ESIONPs (15.5 μg dispersed in 100 μL PBS), EXO (200μg dispersed in 100 μL PBS), or ESIONPs@EXO (200 μg dispersed in 100 μL PBS) every two days. The amount of ESIONPs were equal to that of ESIONPs@EXO normalized to the content of Fe. The tumor size and therapeutic efficacy were evaluated based on signal quantification of intraocular tumor imaging at day 15 post-implantation. The body weight of each mouse was recorded on days 0, 7 and 15, respectively, and the survival of mice was monitored throughout the experiment. Mice with tumor diameter of ≥ 15mm or losses in body weight of ≥20% were promptly euthanized according to the Institutional Animal Care and Use Committee (IACUC) policy.[3] *In vivo* fluorescent imaging and biodistribution of ESIONPs@EXO were detected using DiD labeled ESIONPs@EXO (ESIONPs@EXO-DiD). B16 tumor-bearing or OIR mice were intravenously injected with free DiD, EXO-DiD or ESIONPs@EXO-DiD via the tail vein. The biodistribution of ESIONPs@EXO-DiD in main organs (heart, liver, spleen, lungs, and kidneys), tumors and eyeballs were dissected and measured 24 h after administration using IVIS Spectrum Live Imaging System at an Excitation/Emission of 630/670 nm.

***In vitro* MRI and relaxivity measurements.** The MR imaging and relaxivity were tested using a clinical 3 T MR scanner (Siemens). ESIONPs@EXO was diluted in ultrapure water (C_{Fe} = 0, 0.044, 0.088, 0.175, 0.35, 0.7 mmol L^{-1}) and added to the 5 mL centrifugal tubes. The ESIONPs@EXO dispersions were loaded into tubes and positioned at the center of the MRI scanner coil. The corresponding imaging sequence was utilized to measure the MR imaging and relaxivity. The T1 MRI measurement parameters of 3 T MR scanner were determined as follows:

TE = 1.854 ms, TR = 8.403 ms, FOV = 18 × 18 cm^2, matrix = 384 × 384, slice thickness = 3.0 mm. The T2 MRI measurement parameters of 3 T MR scanner were determined as follows: TE = 20, 40, 60, 80, 100 ms, TR = 989.596 ms, FOV = 20 × 20 cm^2, matrix = 384 × 384, slice thickness = 3.0 mm.

Tumor immunofluorescence. The anti-angiogenic and ferroptosis-promoting effects of the ESIONPs@EXO on tumor were evaluated with immunofluorescence. Specifically, the tumor tissues were first fixed in 4% PFA, dehydrated, paraffin embedded, and sectioned at a thickness of 6 μm. The tissue sections were then subjected to immunofluorescence staining: blocked in 5% BSA for 30 min at RT, followed by overnight incubation at 4°C with rabbit CD31 antibody, rabbit 4HNE antibody, mouse F4/80 antibody or mouse GPX4 antibody (diluted 1:200 in PBS and 0.2% BSA). After washing twice with PBS, the sections were incubated with Alexa Fluor 488 goat anti-rabbit IgG or Alexa Fluor 647 goat anti-mouse IgG secondary antibody (diluted 1:1000 in PBS and 0.2% BSA) for 1 h at RT. After washing with PBS, the slides were subjected to counterstaining with DAPI solution (300 mM) for 10 minutes at RT. Coverslips were subsequently mounted onto glass slides using the Permafluor aqueous mounting medium and the Olympus IX81 microscope was utilized for image acquisition. The presence of CD31-positive staining and fluorescent staining intensity of 4HNE and GPX4 was quantified using ImageJ software.

Hematoxylin-eosin (HE) and Ki67 staining. To evaluate the biological safety of ESIONPs@EXO, vital organs (including the heart, liver, spleen, lungs, and kidneys) were extracted from tumor-bearing mice, and subsequently preserved in 4% paraformaldehyde. These tissues were then embedded in paraffin and sectioned into 8 μm slices, which were subsequently

stained with HE using a HE staining kit in accordance with the manufacturer's instructions. The Ki67 staining technique was used to monitor cellular proliferation within tumor tissues. Specifically, tumor sections were subjected to Anti-Ki67 antibody staining, followed by two washes with PBS and subsequent incubation with Alexa Fluor 647 goat anti-rabbit IgG secondary antibody for 1 hour at RT. Subsequently, the sections were subjected to DAPI staining and subsequently visualized under an Olympus IX81 microscope.

Prussian blue staining. Perls Prussian blue staining was used to detect ESIONPs@EXO deposits using Prussian Blue Iron Stain Kit (G1422, Solarbio life sciences). Briefly, the tissue was initially fixed in a 4% paraformaldehyde solution, followed by conventional procedures of dehydration embedding and sectioning at a thickness of 4 μm. Then, the sections underwent dewaxing and were immersed in distilled water for 1 minute. The Perls stain solution was prepared by combining Perl's stain A1 and A2 in a 1:1 ratio, and the sections were immersed in the Perls dyeing solution for 15-30 minutes. Then, the sections underwent a thorough rinsing process with steaming water for 2-5 minutes. Next, the sections were subjected to exposure to the nuclear solid red staining solution, facilitating a light staining of the nucleus for a period of 5-10 minutes. The sections were washed under running water for 1-5 seconds and followed by conventional dehydration and transparency procedures. Ultimately, the sections were sealed with neutral gum and subsequently observed under an Olympus IX81 microscope.

Blood routine examination and blood biochemistry analysis. To evaluate the blood compatibility of ESIONPs@EXO, blood routine and biochemical indexes were analyzed. C57BL/6 mice were employed for the blood routine and biochemical evaluations. The mice were randomly divided into four groups and administered intravenously with PBS, ESIONPs, EXO

and ESIONPs@EXO. After a period of 14 days, blood samples were collected from the heart (100 μL per mouse). The blood biochemical analysis was performed using an LW C400 Mindray automatic biochemical analyzer (Shenzhen Lanyun Medical Equipment Co., Ltd., Shenzhen, China), and routine examination analysis was performed by automatic hematology analyzer (TEK8500 VET, Tecom Science, Jiangxi, China).

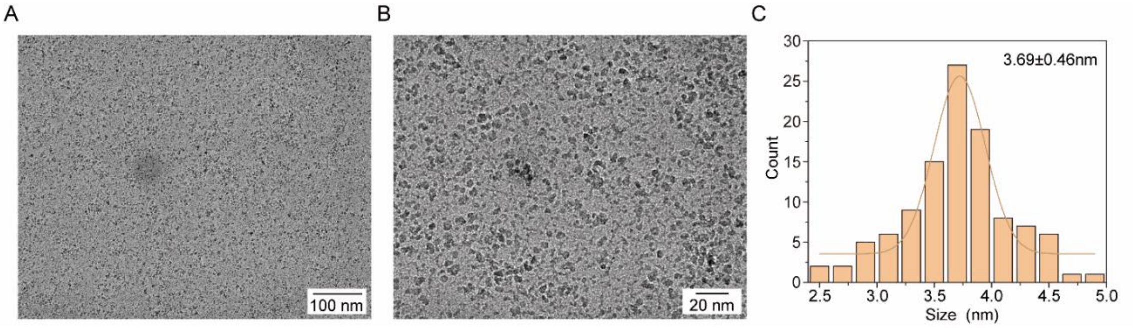

Figure S1. Morphology and the size of the ESIONPs analyzed by TEM. (A) TEM images of ESIONPs. scale bar: 100 nm and 20 nm respectively. (B) Size distribution of ESIONPs.

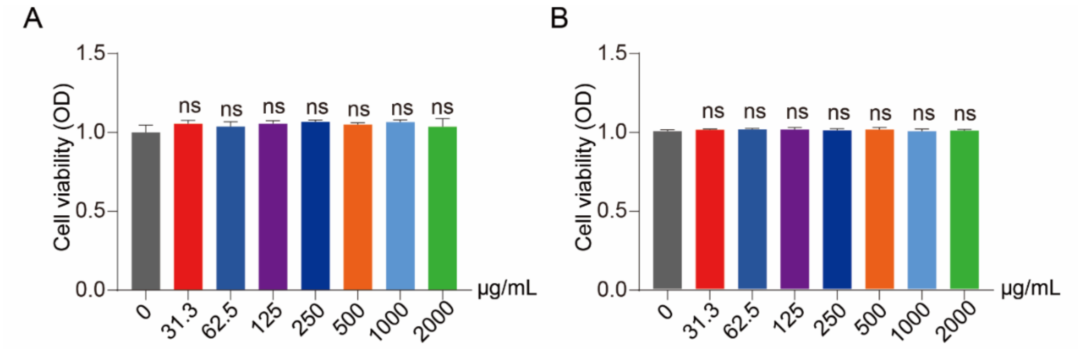

Figure S2. ESIONPs do not affect the viability of the macrophages. ESIONPs did not exhibit significant impact on the viability of BMMs (A) and RAW 264.7 (B) up to a concentration of 2000 μg/mL, Data was presented as means ± SD, n=3, one-way ANOVA.

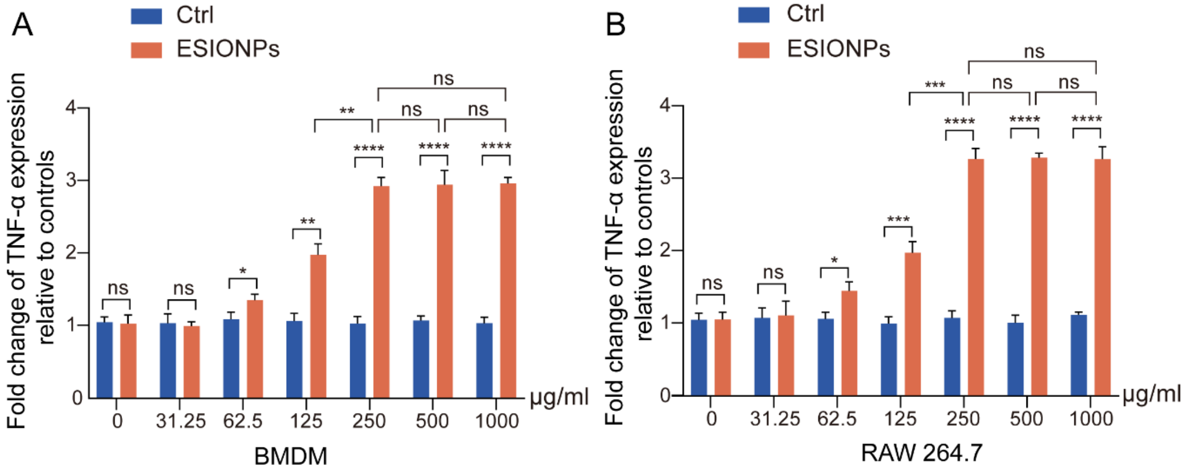

Figure S3. ESIONPs increase TNFα levels in macrophages. ESIONPs could induce pro-inflammatory macrophage activation in both BMMs (A) and RAW 264.7 (B). The levels of M1-associated gene, TNFα, was remarkably upregulated in ESIONPs-treated macrophages with a concentration higher than 62.5 μg/mL. Data was presented as means ± SD, n=3, two-tailed t-test and one-way ANOVA; **P* <0.05; ***P* <0.01; ****P* <0.001; *****P* <0.0001.

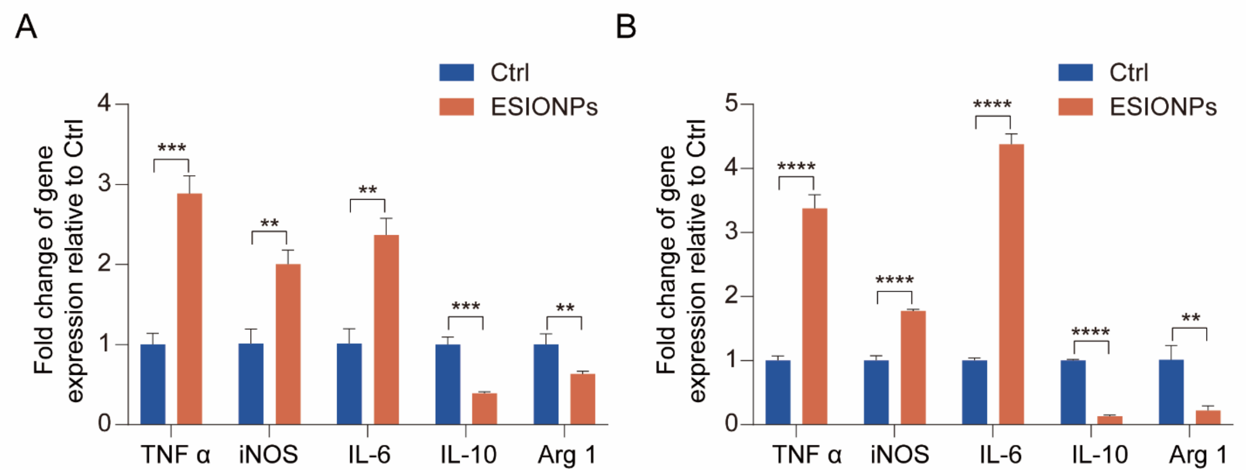

Figure S4. ESIONPs promote M1 macrophage polarization. 250 μg/mL ESIONPs could induce pro-inflammatory macrophage activation in both BMMs (A) and RAW 264.7 (B). The levels of M1-associated genes, such as TNFα, iNOS and IL-6 were remarkably upregulated. Whereas the levels of M2-associated genes, such as IL-10 and Arg1 were downregulated, as measured by quantitative RT-PCR (qRT-PCR). Data was presented as means ± SD, n=3, two-tailed t-test; ***P* <0.01; ****P* <0.001; *****P* <0.0001.

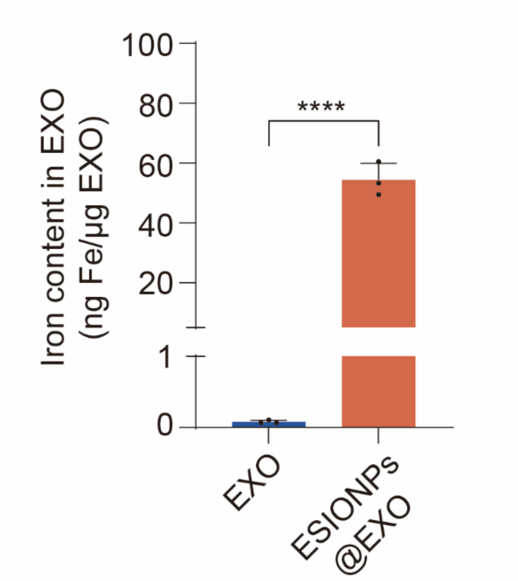

Figure S5. Iron content in ESIONPs@EXO evaluated by ICP-MS. Data was presented as means ± SD, n=3, two-tailed t-test; *****P* < 0.0001.

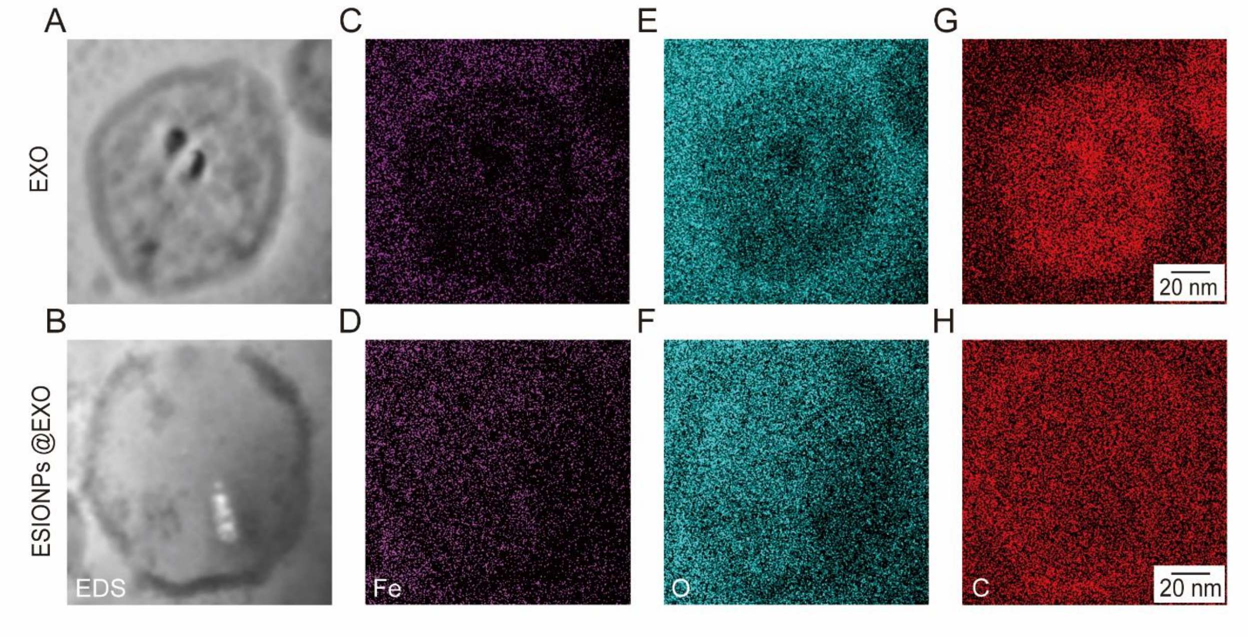

Figure S6. EDS elemental mapping of EXO and ESIONPs@EXO. (A-B) the TEM images of EXO and ESIONPs@EXO. (C-D) the Fe element mapping, (E-F) the O element mapping, (G-H) the C element mapping.

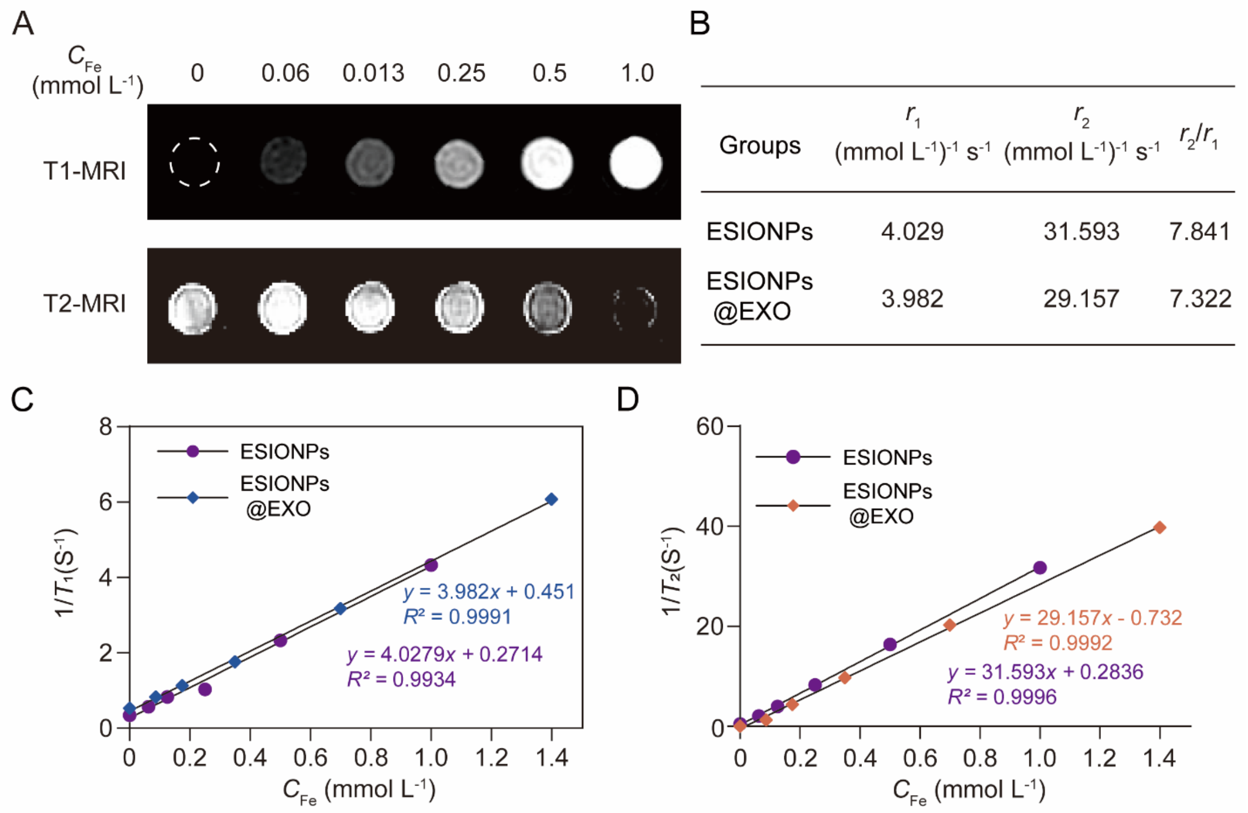

Figure S7. Comparison of relaxation properties of ESIONPs and ESIONPs@EXO. (A) T1 and T2 weighted MR images of ESIONPs (measured on a 3 T MR scanner). (B) Relaxation properties of ESIONPs and ESIONPs@EXO. 1/T1 (C) and 1/T2 (D) relaxation rates of ESIONPs and ESIONPs@EXO at different concentrations.

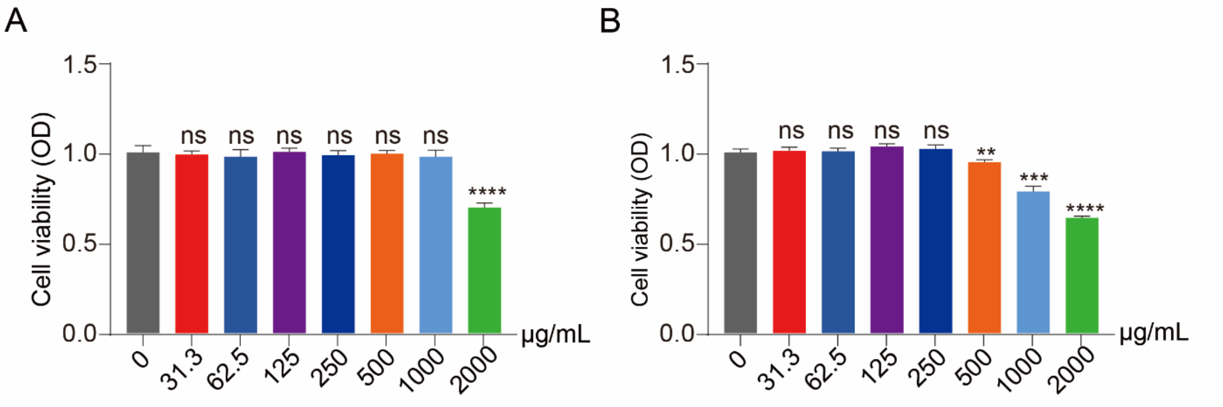

Figure S8. ESIONPs (250 μg/mL) do not affect the viability of C166 and B16 determined by CCK8. ESIONPs did not affect the viability of C166 (A) at a concentration up to 1000 μg/mL, while this value was 250 μg/mL in B16 cells (B) determined by CCK8 analysis. Data was presented as means ± SD, n=3, one-way ANOVA; ***P* <0.01; ****P* <0.001; *****P* <0.0001.

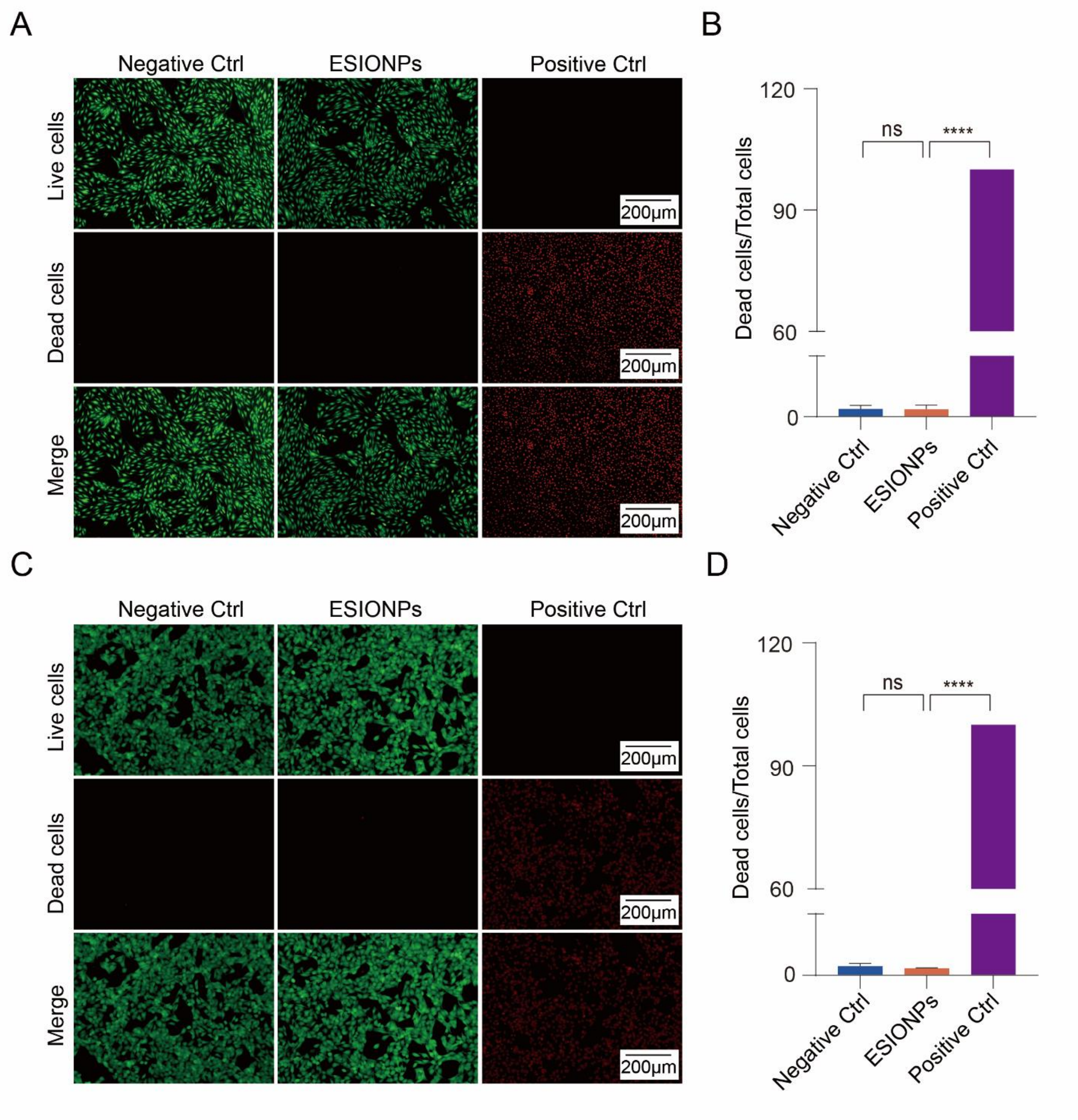

Figure S9. ESIONPs do not cause toxicity in C166 and B16 using dead/live staining. (A) Fluorescent images of C166 after being treated with PBS and ESIONPs. Scale bar: 200 µm. Calcein AM stained live cells (green) and Propidium iodide (PI) stained dead cells (red). (B) Statistical result of C166 cells with dead/live cell staining. (C) Fluorescent images of B16 after being treated with PBS and ESIONPs. Scale bar: 200 µm. Calcein AM stained live cells (green)

and Propidium iodide (PI) stained dead cells (red). (D) Statistical result of B16 with dead/live cell staining. Data was presented as means ± SD, n=3, one-way ANOVA; ****P <0.0001.

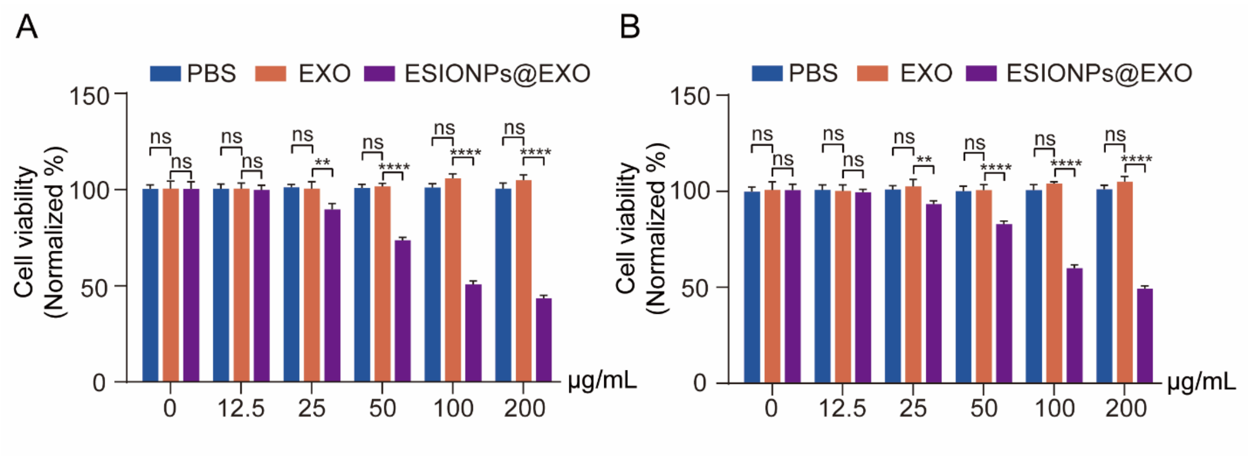

Figure S10. ESIONPs@EXO reduce cell viability *in vitro*. The effect of ESIONPs@EXO on the viability of C166 (A) and B16 (B) up to a concentration of 200 μg/mL, Data was presented as means ± SD, n=3, one-way ANOVA. **P <0.01; ****P <0.0001.

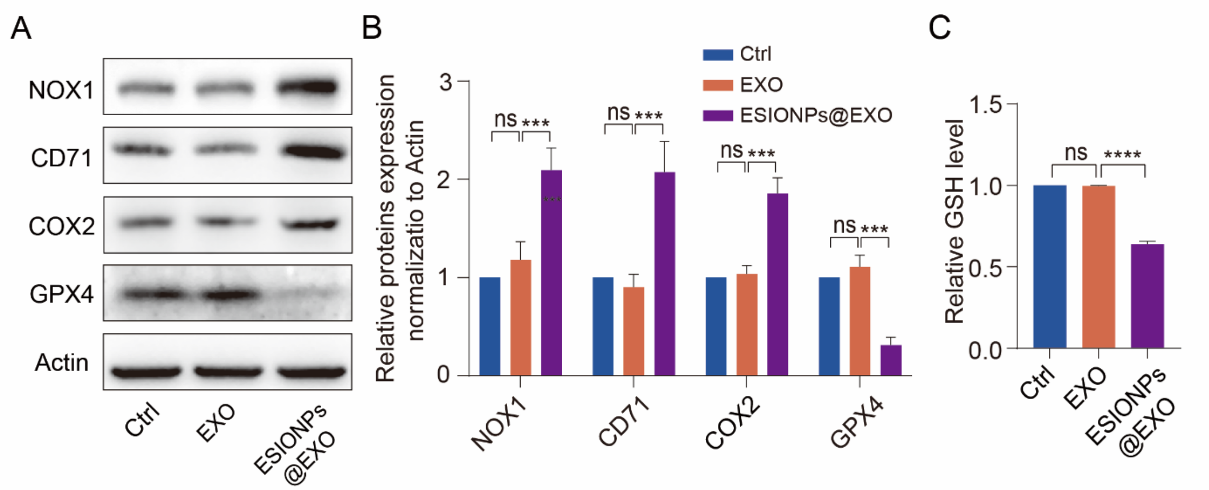

Figure S11. ESIONPs@EXO induce ferroptosis of B16. (A) The protein levels of NOX1, CD71 and GPX4 in ESIONPs@EXO treated B16. (B) Statistical result of the protein levels. (C) Relative GSH level in ESIONPs@EXO treated B16. Data was presented as means ± SD, n=3, one-way ANOVA; ***P <0.001, ****P <0.0001.

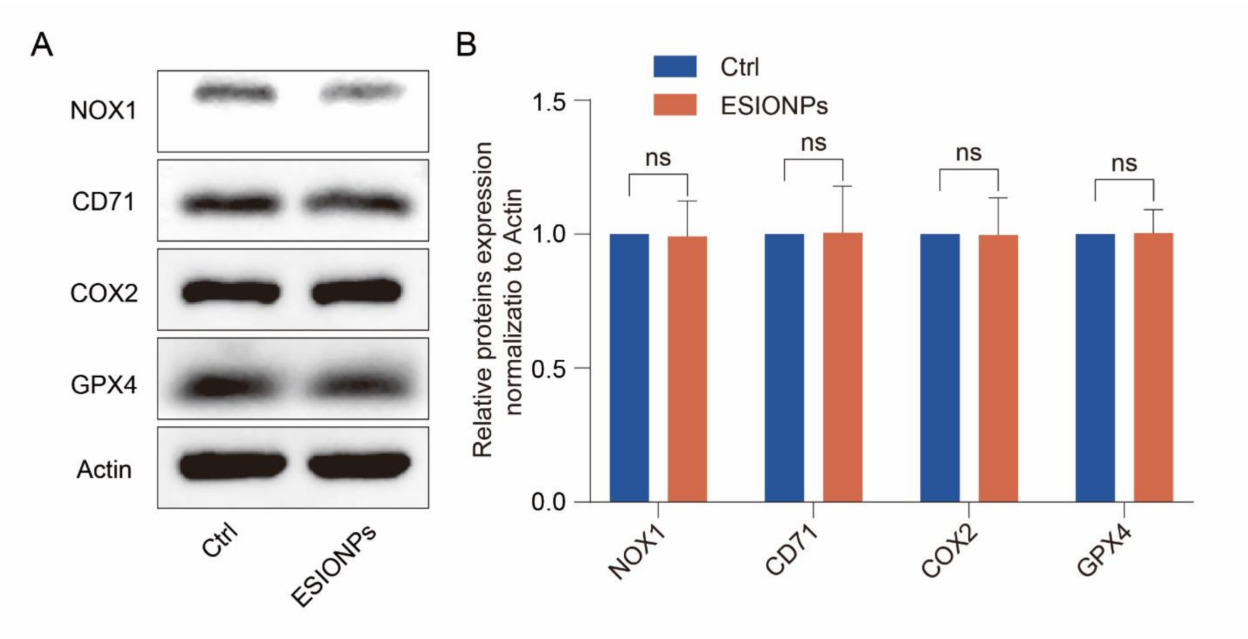

Figure S12. ESIONPs do not induce ferroptosis of C166. (A) The protein levels of NOX1, CD71, COX2 and GPX4 in ESIONPs treated C166. (B) Statistical result of the protein levels. Data was presented as means ± SD, n=3, two-tailed t-test.

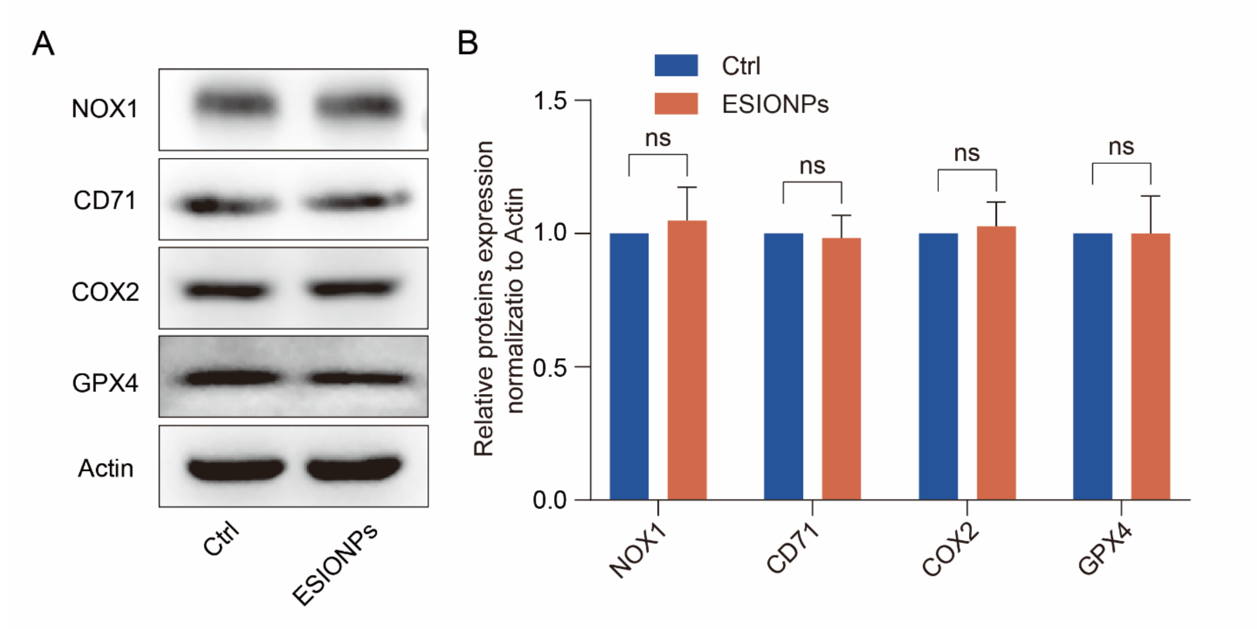

Figure S13. ESIONPs do not induce ferroptosis of B16. (A) The protein levels of NOX1, CD71, COX2 and GPX4 in ESIONPs treated B16. (B) Statistical result of the protein levels. Data was presented as means ± SD, n=3, two-tailed t-test.

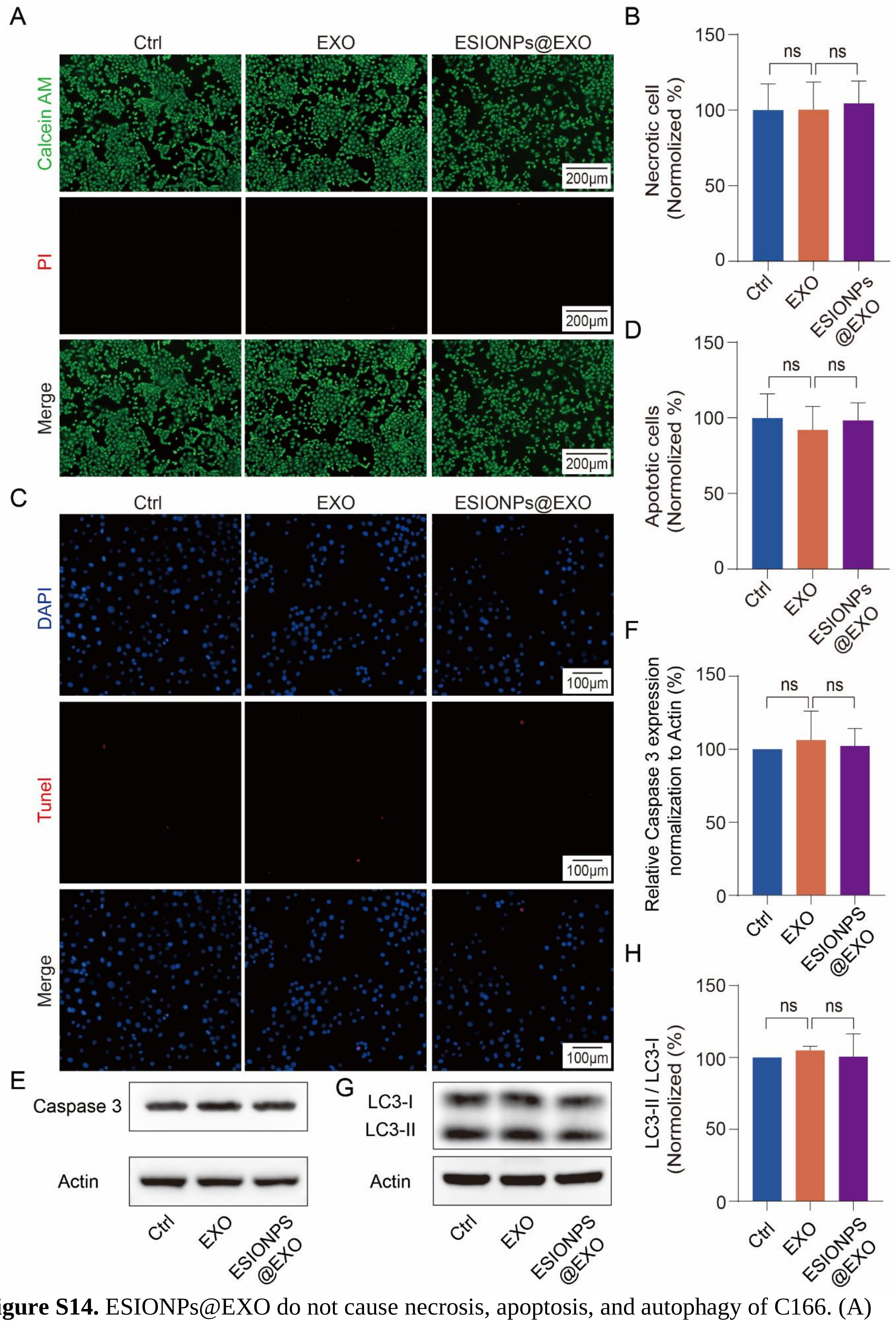

Figure S14. ESIONPs@EXO do not cause necrosis, apoptosis, and autophagy of C166. (A)

Representative images of necrotic cell staining of C166 after being treated with ESIONPs@EXO. Scale bar: 200 μm. Calcein AM-stained live cells was showed in green and PI-stained necrotic cells was showed in red. (B) Statistical result of necrosis in C166. (C) Representative images of TUNEL staining results of C166. TUNEL-positive (red) and Hoechst-positive (blue) cells represent apoptotic and total cells respectively. Scale bar: 100 μm. (D) Statistical result of apoptosis in C166. (E) The protein levels of Caspase 3 in ESIONPs@EXO treated C166. (F) Statistical result of the Caspase 3 levels in ESIONPs@EXO treated C166. (G) The protein levels of LC3 in ESIONPs@EXO treated C166. (H) Statistical result of the LC3 II/I in ESIONPs@EXO treated C166. Data was presented as means ± SD, n=3, one-way ANOVA.

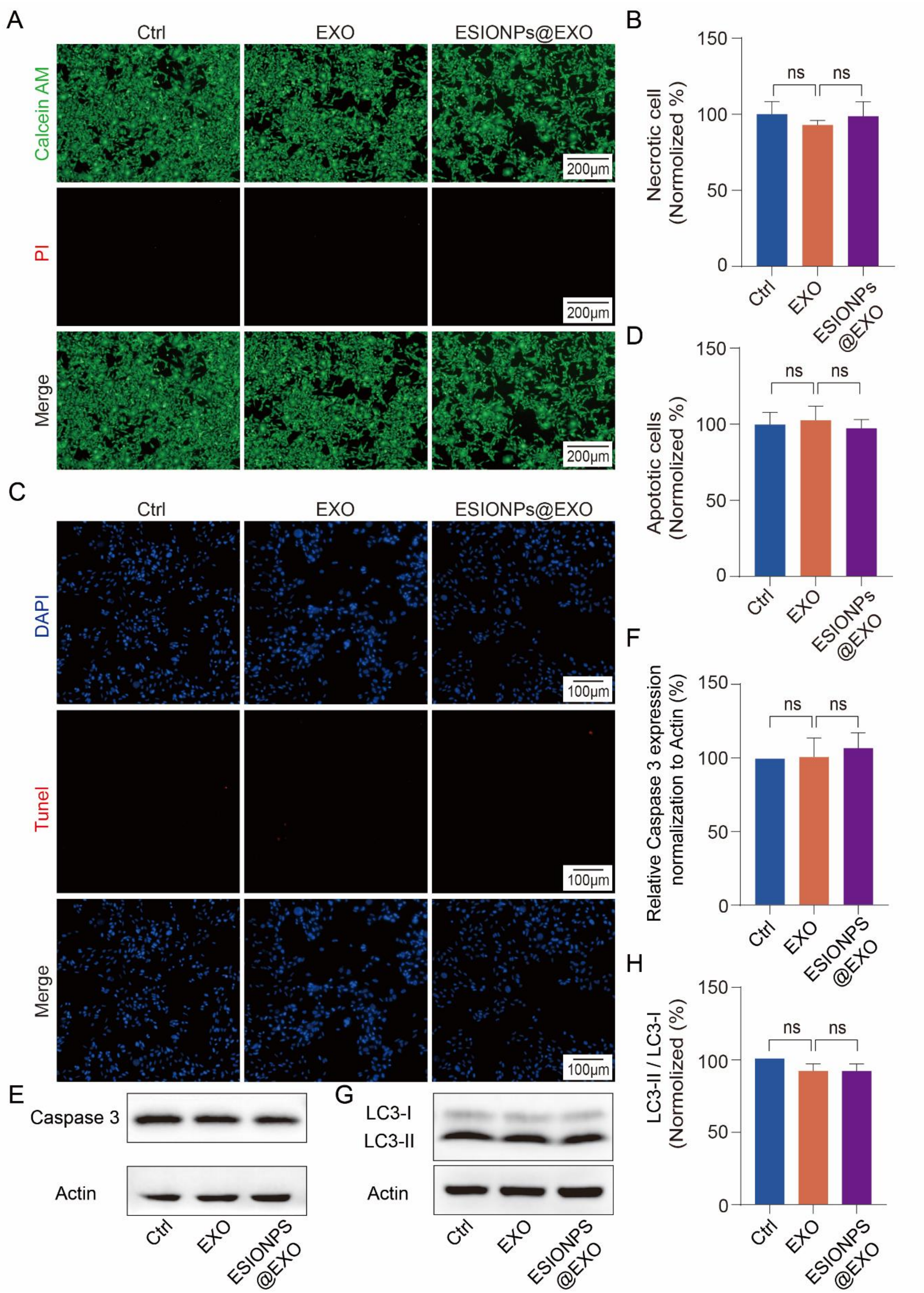

Figure S15. ESIONPs@EXO do not cause necrosis, apoptosis, and autophagy of B16. (A)

Representative images of necrotic cell staining of B16 after being treated with ESIONPs@EXO. Scale bar: 200 μm. Calcein AM-stained live cells was showed in green and PI-stained necrotic cells was showed in red. (B) Statistical result of necrosis in B16. (C) Representative images of TUNEL staining results of B16. TUNEL-positive (red) and Hoechst-positive (blue) cells represent apoptotic and total cells respectively. Scale bar: 100 μm. (D) Statistical result of apoptosis in B16. (E) The protein levels of Caspase 3 in ESIONPs@EXO treated B16. (F) Statistical result of the Caspase 3 levels in ESIONPs@EXO treated B16. (G) The protein levels of LC3 in ESIONPs@EXO treated B16. (H) Statistical result of the LC3 II/I in ESIONPs@EXO treated B16. Data was presented as means ± SD, n=3, one-way ANOVA.

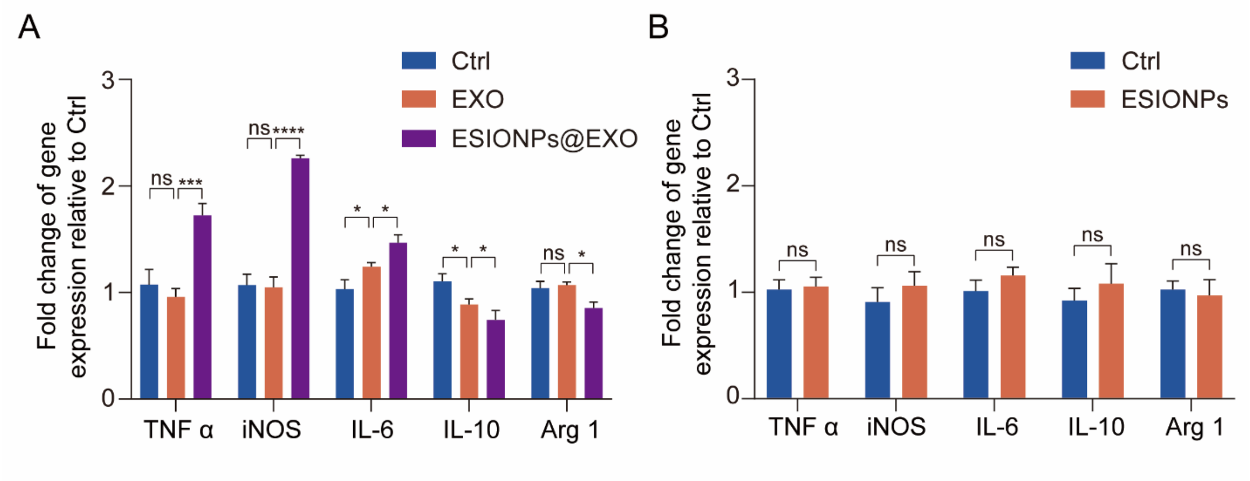

Figure S16. ESIONPs@EXO promote macrophage polarization to M1 phenotype. (A) ESIONPs@EXO (100 μg/mL) could induce pro-inflammatory macrophage activation in BMMs. (B) ESIONPs (7.8 μg/mL) did not induce pro-inflammatory macrophage activation in BMMs. The amount of ESIONPs were equal to that of ESIONPs@EXO (100 μg/mL) normalized to the content of Fe. Data was presented as means ± SD, n=3, two-tailed t-test; *P <0.05; ***P <0.001; ****P <0.0001.

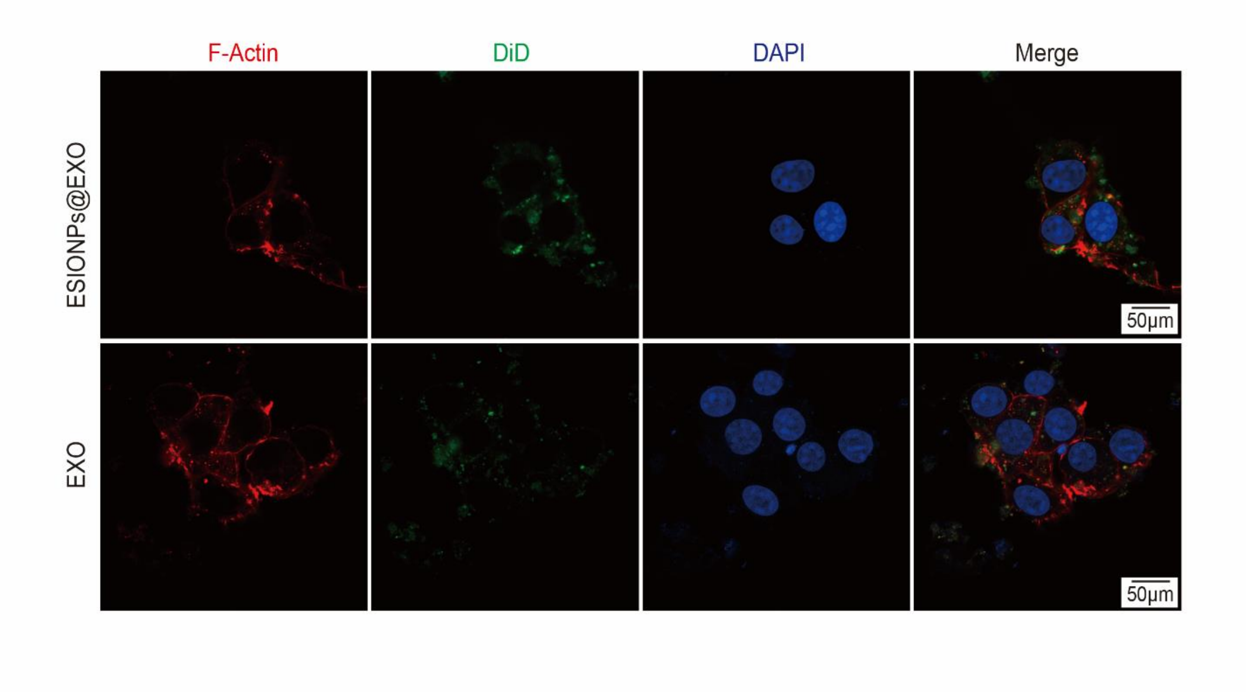

Figure S17. EXO and ESIONPs@EXO were internalized by C166. The DiD-stained ESIONPs@EXO exhibited higher fluorescent intensity compared to the DiD-stained EXO. F-Actin (red), DiD (green) and DAPI (blue). Scale bar: 50 μm.

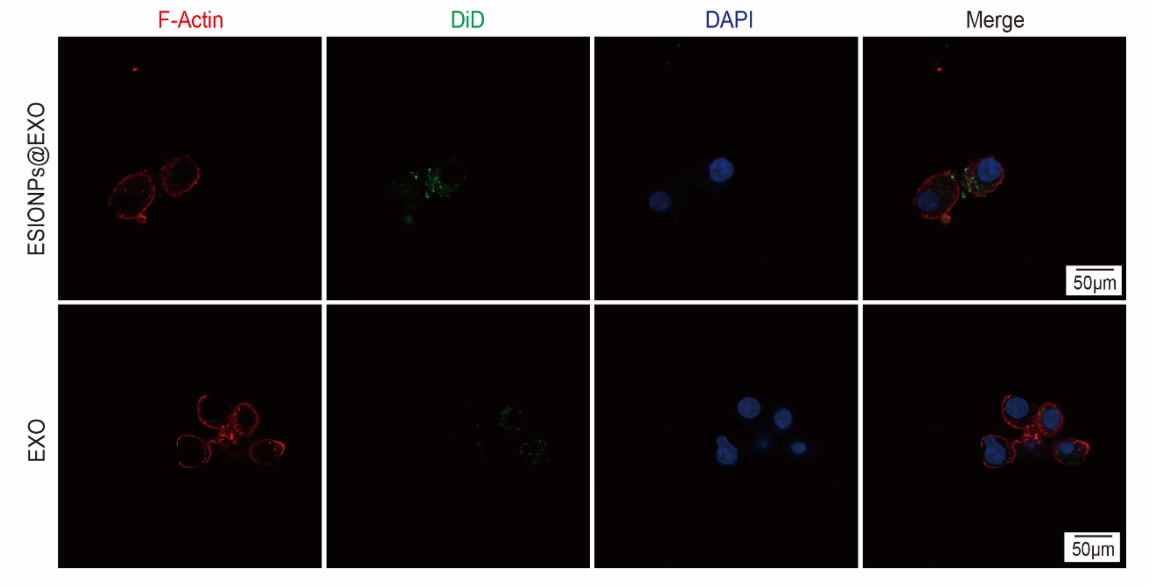

Figure S18. EXO and ESIONPs@EXO were internalized by B16. The DiD-stained ESIONPs@EXO exhibited higher fluorescent intensity compared to the DiD-stained EXO. F-Actin (red), DiD (green) and DAPI (blue). Scale bar: 50 μm.

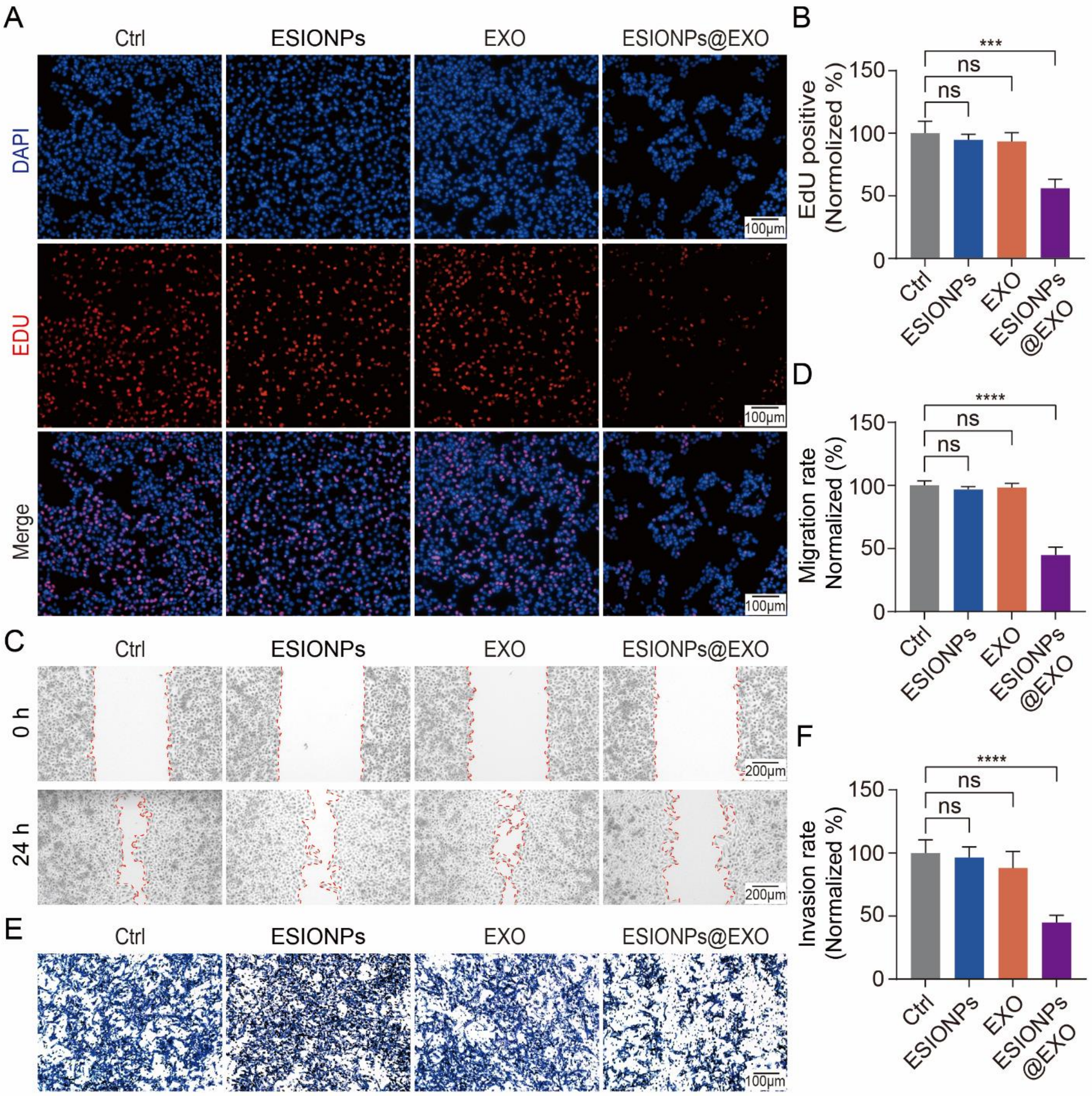

Figure S19. ESIONPs@EXO suppress the growth, migration, and invasion of B16. (A) Representative fluorescent images of EdU incorporation after exposure to ESIONPs@EXO for 24 h. Scale bar: 100 μm. (B) Statistical result of the EdU incorporation. Representative images (C) and statistical images (D) of cell migration after exposure to ESIONPs@EXO for 24 h. Scale bar: 200 μm. Representative images (E) and statistical images (F) of cell invasion after treated with ESIONPs@EXO for 24 h. Scale bar: 100 μm. Data was presented as means ± SD, n=3, one-way ANOVA; ***P <0.001, ****P <0.0001.

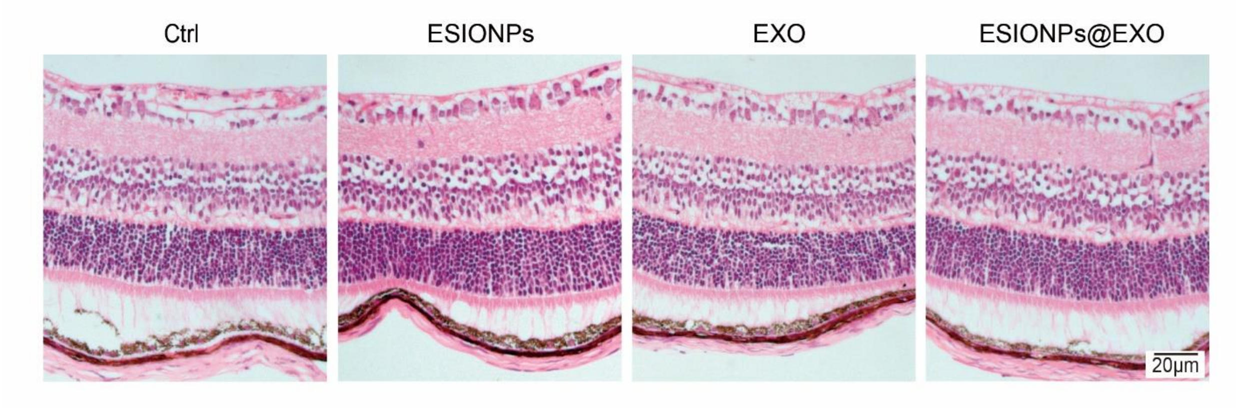

Figure S20. ESIONPs@EXO do not lead to apparent damage to the retina. Representative images of HE staining of mouse retina (P17) with different treatments. Scale bar: 20 μm.

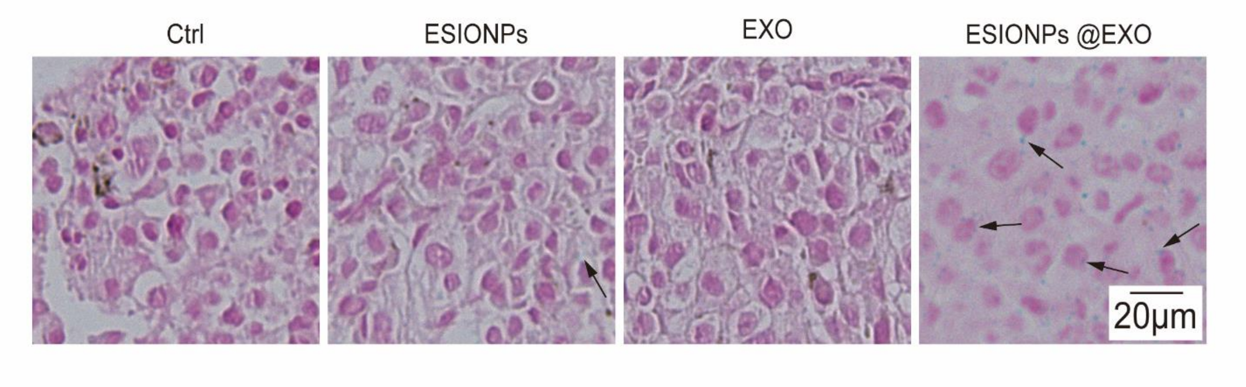

Figure S21. The intracellular accumulation of ESIONPs@EXO stained by Prussian blue in B16 tumor tissues as shown by the black arrow. Scale bars, 20 μm.

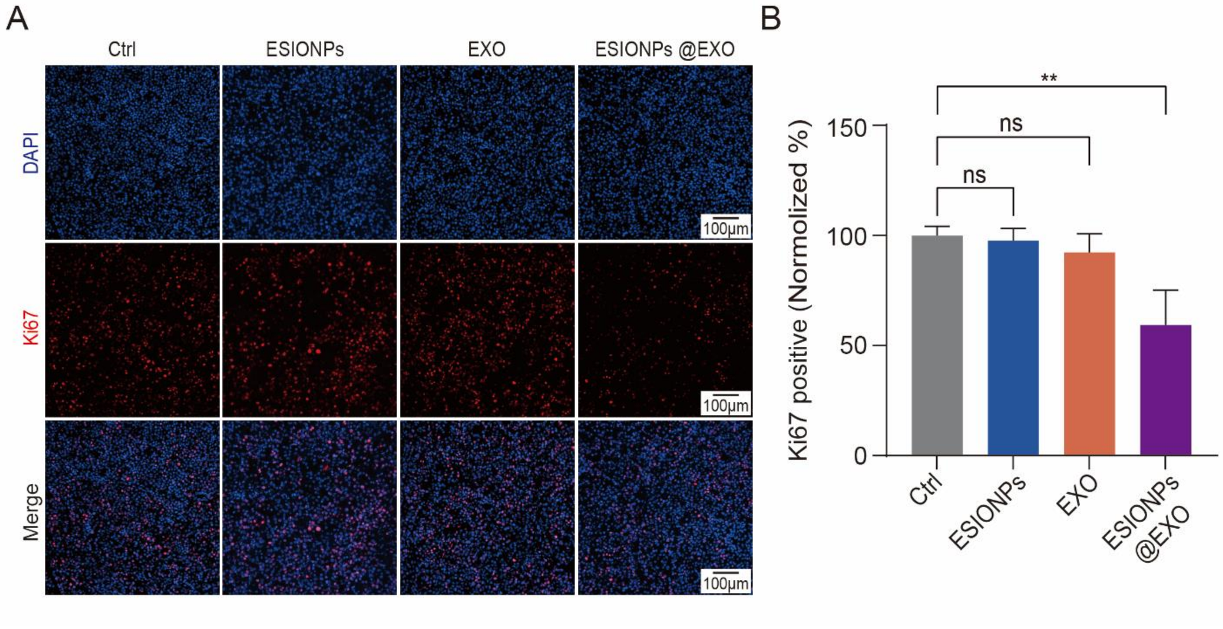

Figure S22. ESIONPs@EXO inhibit B16 proliferation *in vivo*. Representative Ki-67 immunofluorescence staining (A) and statistical images (B) of mice melanoma from xenograft model with different treatments. Scale bar: 100 μm. Data was presented as means ± SEM, n=5, one-way ANOVA; ***P* <0.01.

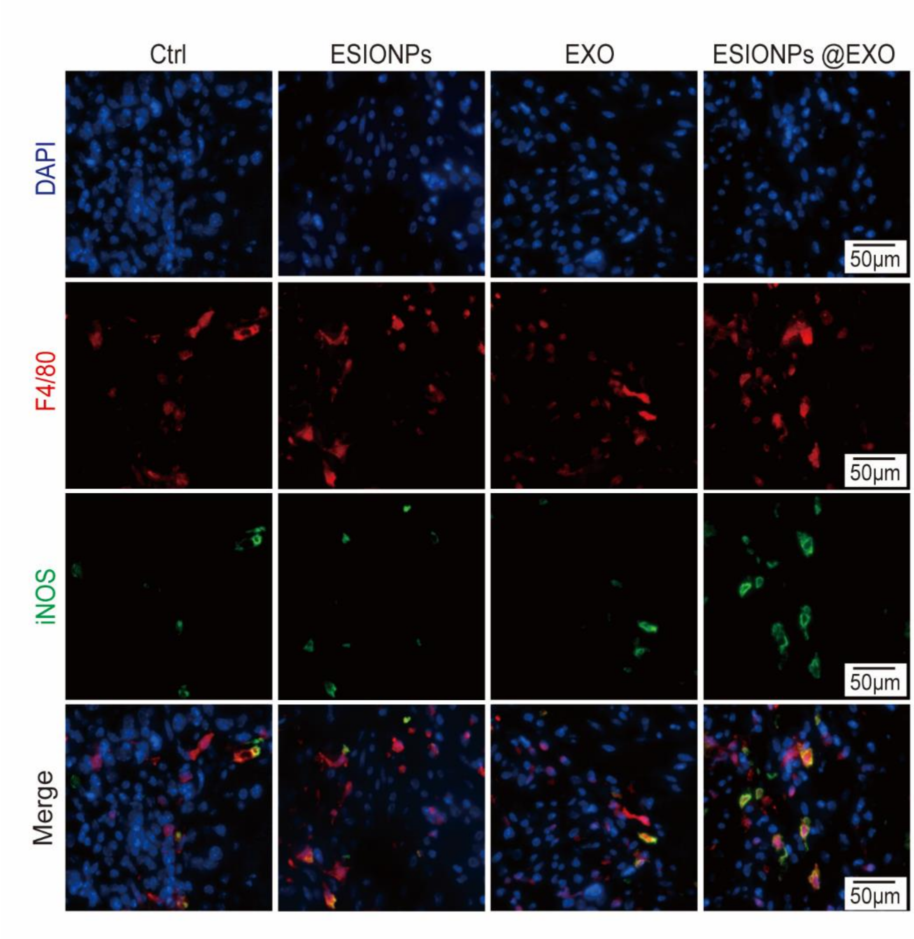

Figure S23. Representative immunofluorescent images of DAPI (blue), iNOS+ (green) and F4/80+ (red) in B16 tumor-bearing C57BL/6J mice. Scale bar, 50 μm.

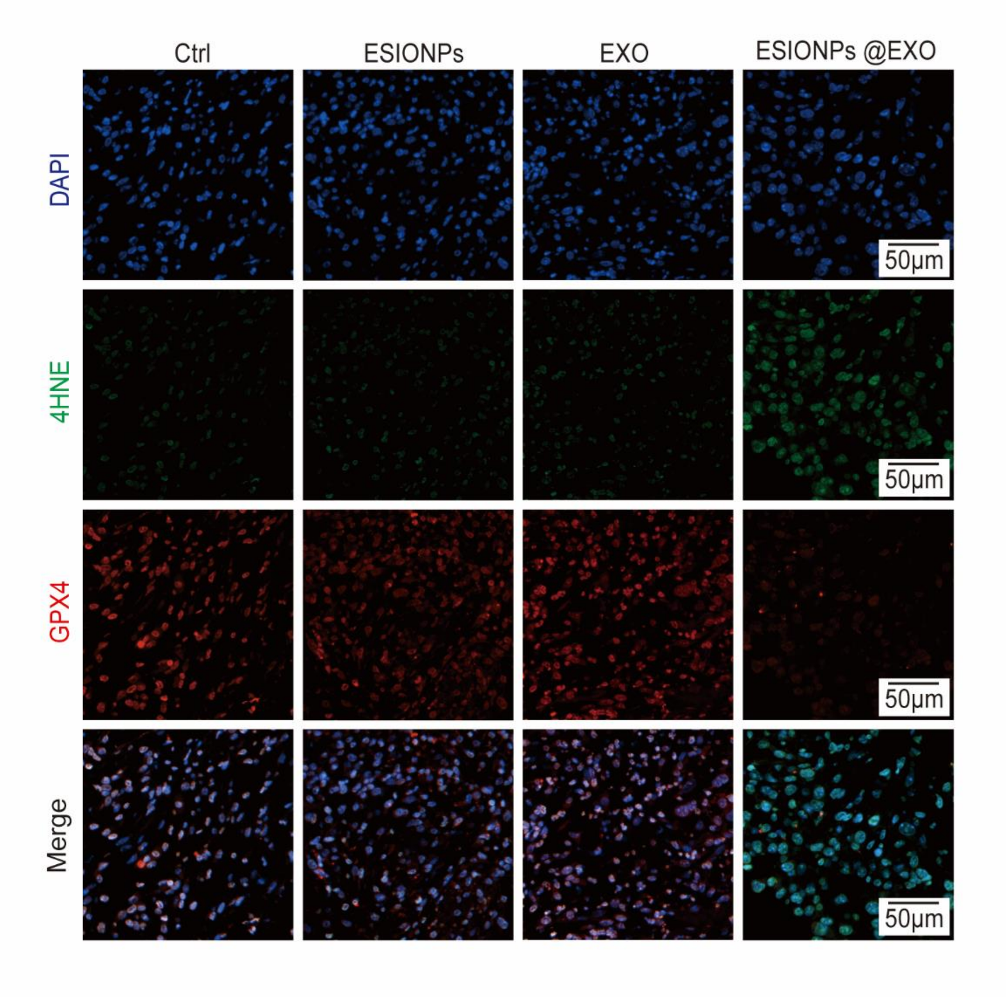

Figure S24. Representative immunofluorescent images of DAPI (blue), 4 HNE (green) and GPX4 (red) in B16 tumor-bearing C57BL/6J mice. Scale bar, 50 μm.

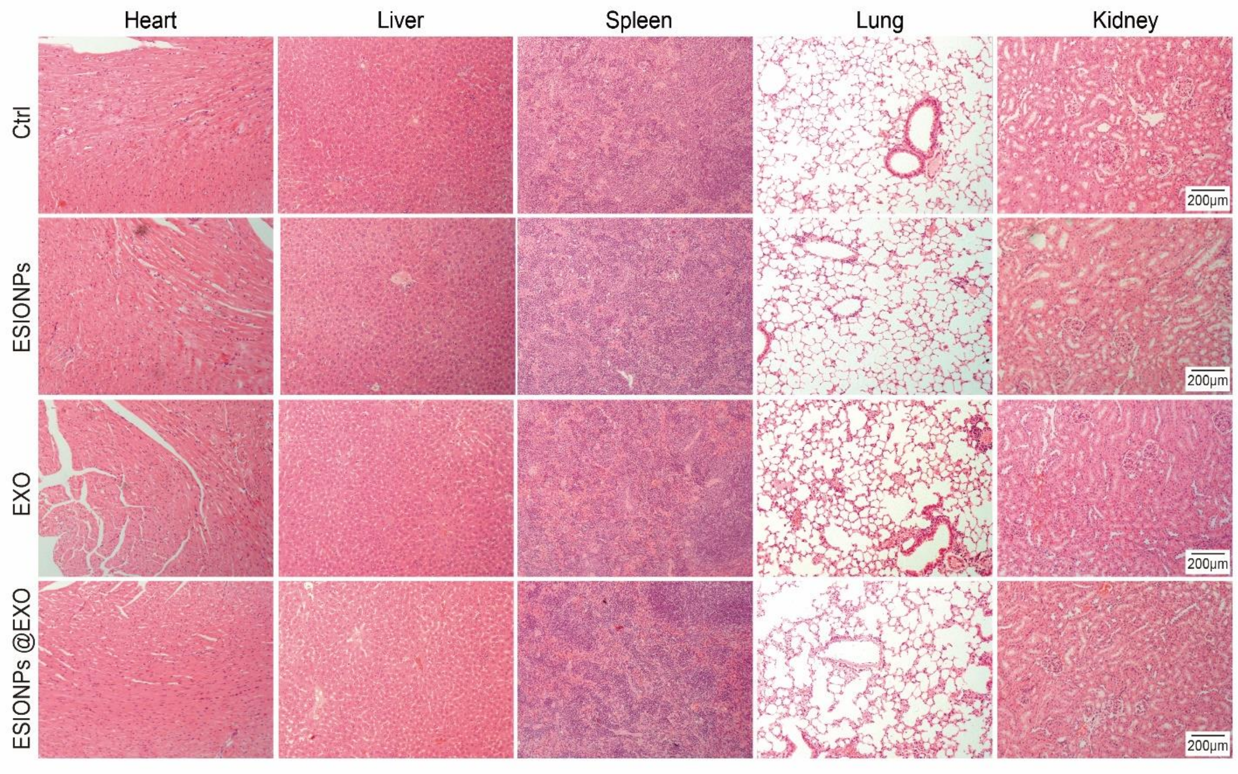

Figure S25. Histological data of the heart, liver, spleen, lung, liver and kidney using HE staining. Scale bar: 200 μm.

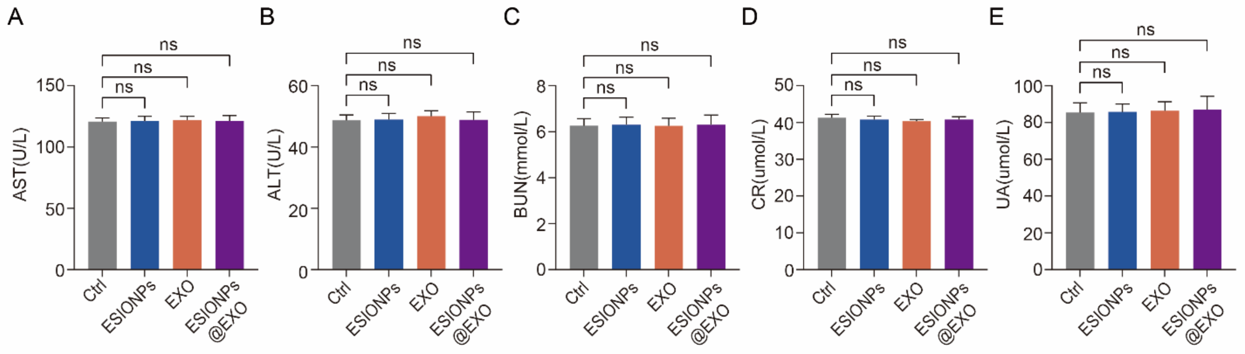

Figure S26. Hepatic and renal function of the blood from mice after treatment with ESIONPs@EXO. None of the indicators changed, including aspartate aminotransferase (AST) (A), alanine aminotransferase (ALT) (B), blood urea nitrogen (BUN) (C), creatinine (CR) (D) and uric acid (UA) (E). The data are presented as the means ± SD. n = 5, one-way ANOVA.

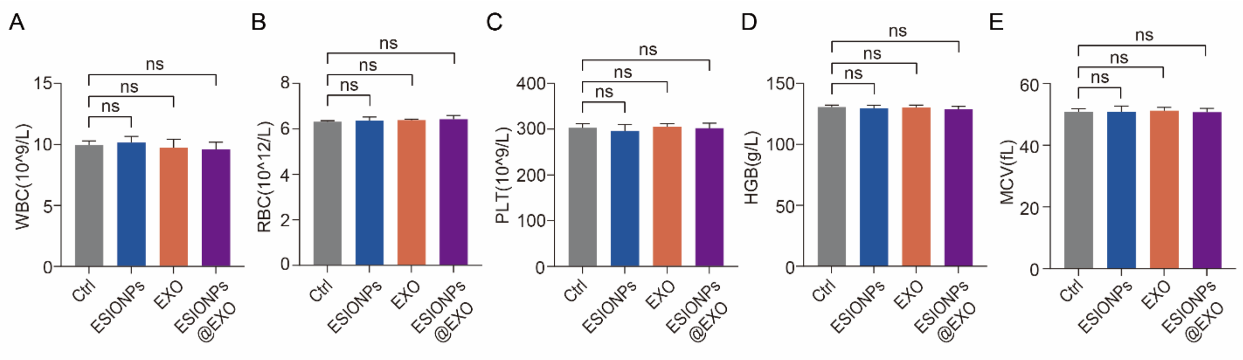

Figure S27. Blood biochemistry of mice after treatment with ESIONPs@EXO. None of the indicators changed, including white blood cell (WBC) (A), red blood cell (RBC) (B), platelet (PLT) (C), hemoglobin (HGB) (D) and mean corpuscular volume (MCV) (E). The data are presented as the means ± SD. n = 5, one-way ANOVA.

Supplementary Tables

Table S1. Primer sequences of candidate genes used in this study

**qPCR primer pairs**	**Sequence (5'-3')**
IL6-Forward	CCACTTCACAAGTCGGAGGCTTA
IL6-Reverse	GCAAGTGCATCATCGTTGTTCATAC
TNFα-Forward	GTCTACTCCCAGGTTCTCTT
TNFα-Reverse	GGTTGACTTTCTCCTGGTATG
iNOS-Forward	CTGCTGGTGGTGACAAGCACATTT
iNOS-Reverse	ATGTCATGAGCAAAGGCGCAGAAC
IL10 -Forward	AGCCTTATCGGAAATGATCCAGT
IL10 -Reverse	GGCCTTGTAGACACCTTGGT
IL-4-Forward	CCCCCAGCTAGTTGTCATCC
IL-4-Reverse	AGGACGTTTGGCACATCCAT
Arg1-Forward	CCCTAATGACAGCTCCTTTC
Arg1-Reverse	CCACACTGACTCTTCCATTC
GAPDH-Forward	AACTTTGGCATTGTGGAAGG
GAPDH-Reverse	ACACATTGGGGGTAGGAACA

Table S2. Summary of antibodies used in this study

Antibody	Dilution	Manufacture (Catalog No.)
**Primary antibodies for immunoblot analysis**		
Beta Actin	1:20000	Proteintech (66009)
Calnexin	1:1000	Proteintech (10427)
Caspase 3	1:1000	Affinity (AF6311)
CCL1	1:1000	Affinity (9910)
CCL3	1:1000	Abclonal (A7568)
CD63	1:1000	Affinity (DF5117)
CD71	1:1000	Proteintech (10084)
CD81	1:1000	Affinity (DF2306)
CD9	1:1000	Affinity (AF5139)
COX2	1:1000	Proteintech (12375)
CX3CL1	1:1000	Abclonal (A14198)
Gapdh	1:1000	Proteintech (10494)
GPX4	1:1000	Proteintech (67763)
IL6	1:1000	Proteintech (21865)
IL9	1:1000	Abclonal (A6630)
LC3	1:1000	Proteintech (14600)
NOX1	1:1000	Proteintech (17772)
TIMP1	1:1000	Abclonal (A1389)
TIMP2	1:1000	Abclonal (A20766)
TNF Alpha	1:1000	Proteintech (60291)
TSG101	1:1000	Proteintech (28283)
**Primary antibodies for Immunofluorescence and immunohistochemistry**		
CD31	1:200	Proteintech (11265)
4HNE	1:200	Bioss (bs6313R)
F4/80	1:200	ServiceBio (GB11027)
iNOS	1:200	R&D Systems (MAB9502)
Ki67	1:500	Proteintech (27309）

(1) Mao, Y.; Li, Y.; Zang, F.; Yu, H.; Yan, S.; Song, Q.; Qin, Z.; Sun, J.; Chen, B.; Huang, X.; Gu, N. Continuous synthesis of extremely small-sized iron oxide nanoparticles used for T1-weighted magnetic resonance imaging via a fluidic reactor. *Sci. China Mater.* **2022,** *65*, 1646-1654.
(2) Song, H.; Guo, T.; Zhao, Z.; Wei, Y.; Luo, H.; Weng, W.; Zhang, R.; Zhong, M.; Chen, C.; Su, J.; Shen, W. Biocompatible PEGylated gold nanorods function as cytokinesis inhibitors to suppress angiogenesis. *Biomaterials* **2018,** *178*, 23-35.
(3) Spina, E.; Simundza, J.; Incassati, A.; Chandramouli, A.; Kugler, M. C.; Lin, Z.; Khodadadi-Jamayran, A.; Watson, C. J.; Cowin, P. Gpr125 is a unifying hallmark of multiple mammary progenitors coupled to tumor latency. *Nat. Commun.* **2022,** *13*, 1421.
